# DNA Methylation Changes Induced by Cold in Psychrophilic and Psychrotolerant *Naganishia* Yeast Species

**DOI:** 10.3390/microorganisms8020296

**Published:** 2020-02-20

**Authors:** Benedetta Turchetti, Gianpiero Marconi, Ciro Sannino, Pietro Buzzini, Emidio Albertini

**Affiliations:** Department of Agricultural, Food and Environmental Sciences, University of Perugia, Borgo XX Giugno, 74, 06121 Perugia, Italy; gianpiero.marconi@unipg.it (G.M.); ciro.sannino@unipg.it (C.S.); pietro.buzzini@unipg.it (P.B.); emidio.albertini@unipg.it (E.A.)

**Keywords:** cold-adapted yeasts, cold stress response, *Naganishia albida*, *Naganishia antarctica*

## Abstract

The involvement of DNA methylation in the response to cold stress of two different yeast species (*Naganishia antarctica*, psychrophilic, and *Naganishia albida*, psychrotolerant), exhibiting different temperature aptitudes, has been studied. Consecutive incubations at respective optimum temperatures, at 4 °C (cold stress) and at optimum temperatures again, were performed. After Methylation Sensitive Amplified Polymorphism (MSAP) fingerprints a total of 550 and 423 clear and reproducible fragments were amplified from *N. antarctica* and *N. albida* strains, respectively. The two *Naganishia* strains showed a different response in terms of level of DNA methylation during cold stress and recovery from cold stress. The percentage of total methylated fragments in psychrophilic *N. antarctica* did not show any significant change. On the contrary, the methylation of psychrotolerant *N. albida* exhibited a nonsignificant increase during the incubation at 4 °C and continued during the recovery step, showing a significant difference if compared with control condition, resembling an uncontrolled response to cold stress. A total of 12 polymorphic fragments were selected, cloned, and sequenced. Four fragments were associated to genes encoding for elongation factor G and for chitin synthase export chaperon. To the best of our knowledge, this is the first study on DNA methylation in the response to cold stress carried out by comparing a psychrophilic and a psychrotolerant yeast species.

## 1. Introduction

Yeasts are mainly known for their importance in food and beverage fermentations, but they are also naturally distributed in a large variety of environments, controlling crucial ecological processes. Due to their saprotrophic status, yeasts are known to be primary decomposers, but they can also act as mutualists, competitors, parasites, or pathogens [1,2]. Yeasts showed a wide distribution in terrestrial ecosystems: they were found from the upper levels of the atmosphere, to the phyllosphere, in hot and dry deserts, the deepest parts of the oceans, and in ancient glacial ice [3,4,5]. Cold habitats, representing over 80% of the Earth’s total environments, were explored for yeasts occurrence since the 1960s [6,7,8]. In the course of the past decades, a number of yeasts species were isolated from worldwide cold habitats and many new species were described [7,8,9]. 

Microorganisms (including yeasts) that successfully live and grow in cold environments are usually defined psychrophiles (otherwise labeled as obligate psychrophiles), whereas the organisms that tolerate low temperature are labeled psychrotolerants (or facultative psychrophiles). Conventionally, this classification is based on limits of temperature allowing cell growth. In particular, whereas psychrophiles show an optimum growth temperature of 15 °C or lower and a maximum growth temperature not higher than 20 °C, psychrotolerants exhibit a wider range of growth temperature (optimum at 20–30 °C) and are able to survive or even duplicate until 0 °C [10]. 

Psychrophilic microorganisms sharing cold ecosystems are frequently subjected to a series of concurrent environmental stresses. Often, low temperature is associated to extremely high pressure, low water activity (Aw), oligotrophic conditions, and exposition to strong UV radiation [6,10]. 

Modulation of gene expression as responses to cold stress has been studied so far in a few microorganisms, prevalently prokaryotes [11]. Cold shock response (CSR) in *Escherichia coli* was largely studied through the analysis of cold shock genes expression. Among CS gene products nucleic acid-binding proteins were showed to be the prevalent. These proteins are involved in DNA replication, supercoiling and transcription, RNA degradation and translation, and genesis of the ribosome. Other CS proteins associated to cold shock and expressed during abrupt reduction of temperature comprise protein chaperones (TF and Hsc66), two proteins implicated in trehalose (protective macromolecules) synthesis, and two involved in lipid biosynthesis and cellular metabolism [12]. The induction of these proteins indicates that CSR is involved in developing different strategies for improving their life (or even survival) chances at near-zero temperature, such as supporting in protein folding, enhance membrane fluidity, and accumulate macromolecules in order to obtain a protective effect against the low temperature [6,13,14,15,16,17,18,19,20]. Cold-stressed response of a representative bacterium of food processing industry (*Lactobacillus plantarum* K25 isolated from kefir) was also analyzed using iTRAQ proteomic method. A large number of proteins differentially expressed at 10 °C and 37 °C were identified, all involved in carbohydrates, aminoacids, and fatty acid biosynthesis (downregulated) and DNA repair system, transcription, and translation (upregulated) [21]. In cyanobacteria the expression of ~100 genes was associated to cold stress. Some of these genes were connected to defend cellular functions by adjustment of membranes composition, as well as transcriptional and translational modulation. Additionally some of the cold-induced genes were associated to a two-component regulatory system, consisting of histidine kinase Hik33 and response regulator Rre26 [22]. Approaches based on global transcript profiling using DNA microarray analysis [23,24], genome-wide screening of mutants [25], differential mRNA performance [26], but also transcriptomic analysis approach have been used to study cold-shock responses in yeasts [23,27,28]. Two strains of the basidiomycetous psychrophilic species *Mrakia blollopis* have been investigated through capillary electrophoresis-time-of-flight mass spectrometry (CE-TOFMS) in order to detect their metabolomic response to cold stress under sub-zero temperature: the accumulation of high levels of TCA cycle metabolites, lactic acid, aromatic amino acids, and polyamines were interpreted as CSR [20]. Recently the cold-adaptation strategies of *Glaciozyma antarctica* PI12 were investigated using a transcriptomic analysis approach. Freeze stress (0 °C and −12 °C) showed 168 genes differentially expressed. *G. antarctica* PI12 exhibited some common adaptation strategies with other yeasts such as *Saccharomyces cerevisiae* and *Mrakia* spp., but also unique mechanisms, including the production of antifreeze protein to prevent ice-crystallization inside and outside the cell, and the constitutively expression of several molecular chaperones, detoxifiers of reactive oxygen species (ROS), and transcription and translation genes [29].

Methylation of cytosine to 5-methylcytosine (m5C) is an important epigenetic modification widely detected in bacteria, plants and mammalian cells [30,31]. Several studies have linked this epigenetic regulation to gene expression in development and environmental cues [32]. The presence of such DNA modification in some organisms is controversial and some authors hypothesized the complete absence or very low presence of m5C in some organisms including model species, such as *Caenorhabditis elegans*, *Neurospora crassa*, *Dictyostelium discoidium*, *Schistosoma mansoni*, and *Drosophila melanogaster* [33,34,35,36,37,38,39]. 

Currently, DNA methylation has been studied in a few yeasts species. Some studies postulated the absence of this epigenetic modification while some others demonstrated the existence of a variable degree of DNA methylation [30,39,40,41,42]. Despite these investigations, so far, no studies have been conducted in order to unravel the role of DNA methylation in psychrophilic and psychrotolerant yeasts in the response to the stress induced by low temperatures.

The aim of this study was (i) to investigate the existence of DNA methylation in two yeast species belonging to the same genus (the psychrotolerant *Naganishia albida* and the psychrophilic *Naganishia antarctica*) employing the reliable and well-characterized Methylation Sensitive Amplified Polymorphism (MSAP) method and (ii) to assess its possible involvement as adaptation mechanism in response to cold stress.

## 2. Materials and Methods 

### 2.1. Yeast Strains

The yeast strains used in the present study are *Naganisha albida* DBVPG 10064 (psychrotolerant) and *Naganisha antarctica* DBVPG 5271 (psychrophilic), both conserved in the Industrial Yeast Collection, DBVPG of the University of Perugia, Perugia, Italy (www.dbvpg.unipg.it). They were preliminarily selected as model strains: *N. albida* DBVPG 10064 was isolated from supraglacial sediment of Miage glacier, Mont Blanc, Italian Alps, whereas *N. antarctica* DBVPG 5271 was isolated from morainic soil of the same glacier characterized by temperature fluctuations (day and night but also summer and winter) [43]. Both strains were routinely maintained in physiological inactive/immobilized state (−80 °C). Working cultures were grown on YEPG (yeast extract 10 g L^−1^, peptone 10 g L^−1^, glucose 20 g L^−1^, agar 15 g L^−1^) agar slants at 25 °C (*N. albida*) or 20 °C (*N. antarctica*).

### 2.2. Determination of the Optimum Temperature

Forty-eight-hour-old cultures of both strains were inoculated in Petri dishes containing YEPG agar as serial diluted suspensions (10 fold less concentrated) starting from a concentration calibrated to A580 = 0.8 (average cell concentration = 10^7^ cells/mL), incubated at 4, 10, 15, 20, 25, and 30 °C and then inspected for growth. After 7 days, the temperature at which the production of the highest quantity of biomass was found at the most diluted suspension was considered as the optimal.

### 2.3. Yeasts Growth

**Figure 1:**
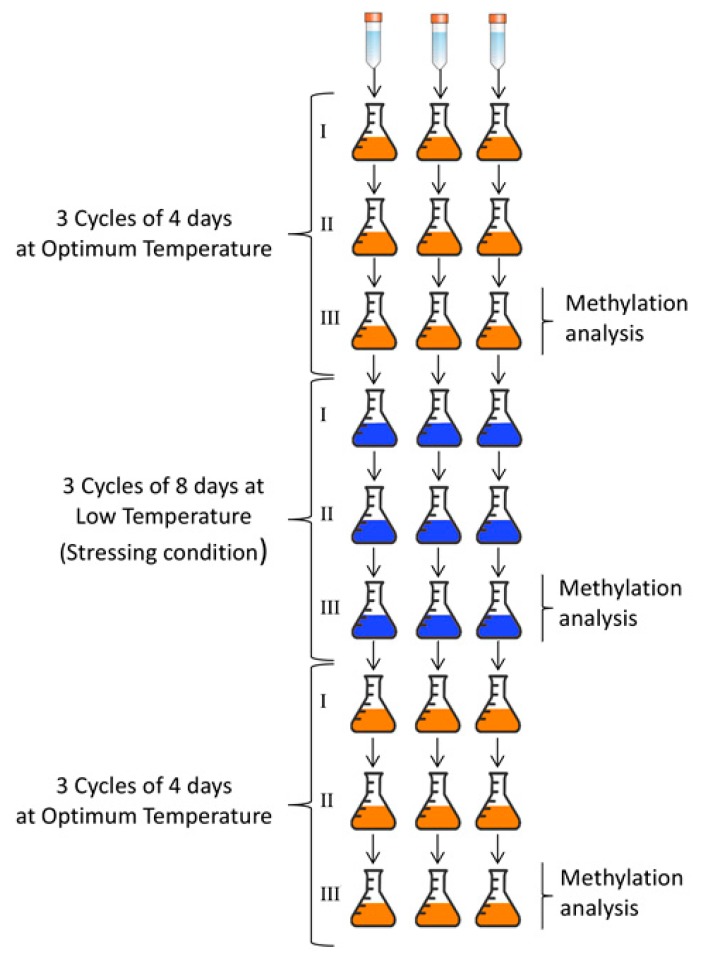
Experimental research design. The same procedure was applied in triplicate to both *N. antarctica* DBVPG 5271 and *N. albida* DBVPG 10064.

Calibrated (A580 = 0.8, which was considered an average cell concentration = 10^7^ cells/mL) suspensions of 48 h-old cells of *N. albida* DBVPG 10064 and *N. antarctica* DBVPG 5271 grown at the respective optimum temperatures were obtained. For each strain 3 Erlenmeyer flasks containing 50 mL of YEPG broth were inoculated using 1 mL of the obtained suspensions. Four other additional serial diluted suspensions (10-fold less concentrated) were obtained for each strain starting from the calibrated one. Each suspension was inoculated in Petri dishes containing YEPG agar medium as single spot (10 µL). Flasks and Petri dishes were incubated at the strains’ respective optimum temperature (25 °C for *N. albida* DBVPG 10064 and 20 °C for *N. antarctica* DBVPG 5271) until reaching the stationary phase (Figure 1); flasks were incubated in an orbital shaker (110 rpm). After incubation, the individual steps described above were repeated twice, using the cells derived from the development of the previous inoculum as biomass for the formation of the suspension. The biomass derived from the third inoculation at optimum temperature was stored at −20 °C for the subsequent DNA extraction (Figure 1), and partially used to obtain 3 further seeding and incubation cycles at 4 °C until reaching the stationary phase (Figure 1). The biomass derived from the third inoculation at 4 °C was stored at −20 °C for the subsequent DNA extraction, and partially used to obtain 3 further seeding and incubation cycles at respective optimum temperature until reaching the stationary phase. The biomass derived from the third inoculation at optimum temperature was partially stored at −20 °C for the subsequent DNA extraction. As shown in Figure 1, summarizing cells for DNA extraction were sampled three times: at the end of the third cycle of incubation at optimum temperature (control condition), at the end of the third cycle of incubation at 4 °C (cold stress), and at the end of the third cycle of the second incubation at optimum temperature (recovery from cold stress). All the tests were performed in triplicate (three independent biological repetitions). Yeast growth was monitored at the end of each cycle using dry mass weight and viable cell counts on YEPG agar plates [44]. 

### 2.4. DNA Methylation Analysis

To evaluate the state of methylation in cells completely adapted to the settled temperature conditions, the first and second cycles of each step of incubation were not considered for DNA extraction and subsequent study of methylation events. DNA was extracted as reported in Turchetti et al. [45]. Methylation Sensitive Amplified Polymorphism (MSAP) technique was applied in *N. albida* DBVPG 10064 and *N. antarctica* DBVPG 5271 at different temperatures: optimum and cold stress, following the procedure of Marconi et al. [46]. A total of 12 primer combinations were used for selective amplification (Table S3). Each amplified sample was separated on an ABI 3130 XL Genetic Analyzer (Applied Biosystems, Foster City, CA, USA). Amplified fragments were divided into four types based on the presence or absence of bands, which resulted from the differential sensitivity of the fragments to digestion by *Msp*I and *Hpa*II. Type I represents the presence of bands in both enzymes combinations, i.e., *Eco*RI/*Hpa*II and *Eco*RI/*Msp*I, type II bands appeared only in *Eco*RI/*Hpa*II but not in the *Eco*RI/*Msp*I, type III generated bands in *Eco*RI/*Msp*I but not in the *Eco*RI/*Hpa*II, and type IV represents the absence of bands following both enzyme combinations. Type II indicates the hemimethylated state of DNA that results from methylation in one DNA strand, but not in its complementary strand [47]. Type III represents the case of full CG (internal cytosine) methylation, whereas type IV is the case of full methylation at both cytosines.

The procedure of isolating polymorphic fragments was described in Marconi et al. [46]. Polymorphic fragments selected for being differentially methylated were excised from acrylamide gels. In particular, to focus on genes directly involved in cold stress response, the selected fragments were the ones showing an opposite behavior in control and stress condition and the same behavior between control and recovery (when the optimum conditions were restored), e.g., control: methylated; stress: demethylated; recovery: methylated. Each selected fragment was re-amplified and cloned using the TOPO TA cloning kit for sequencing (Thermo Fisher Scientific, Waltham, US). Ten colonies for each transformation were sequenced with the BigDye^®^ Terminator v3.1 Cycle Sequencing Kit, Applied Biosystems Foster City, CA, USA) on an ABI 3130xl Genetic Analyzer sequencer (Applied Biosystems Foster City, CA, USA). Differentially methylated fragments (DMF) sequences were used as queries in a BLAST search.

## 3. Results

### 3.1. Yeasts Growth

The growth of *N. albida* DBVPG 10064 and *N. antarctica* DBVPG 5271, monitored at the end of each cycle (Figure 1), showed a well-defined trend. 

**Figure 2:**
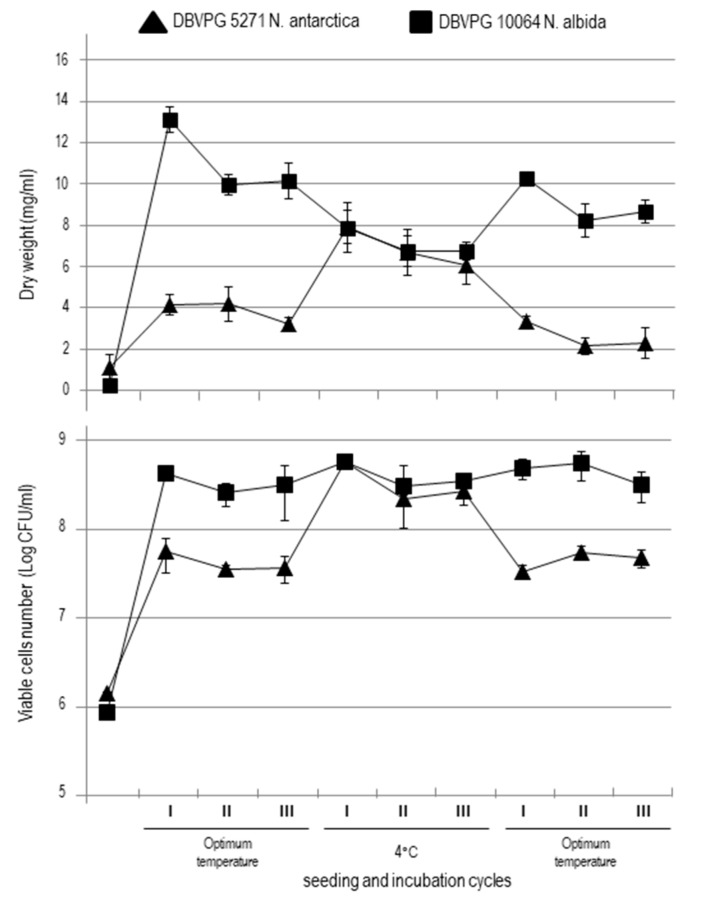
Time course of cell growth of *N. antarctica* DBVPG 5271 and *N. albida* DBVPG 10064 during the seeding and incubation cycles. Dry weight and viable cell number were shown. Error bars indicate the SEM.

Dry mass weight and viable cell counts (Figure 2) gave similar results. At optimum temperatures, the two strains showed different growth levels: *N. albida* grown at 25 °C gave a number of cells one logarithmic unit higher than that exhibited by *N. antarctica* at 20 °C. 

Interestingly, the biomass of the two strains incubated at 4 °C (cold stress) was not significantly different in any of the three cycles. In particular, *N. albida* dry mass decreased, whereas *N. antarctica* increased the total number of cells and dry mass (Figure 2). Finally, when the cultures were incubated back at their individual optimum temperatures, the number of cells of *N. antarctica* decreased reaching the quantity shown in the first three cycles of incubation while the number of cells of *N. albida* remained stable in all the three assayed conditions, showing only some fluctuation when dry mass was considered. 

**Figure 3:**
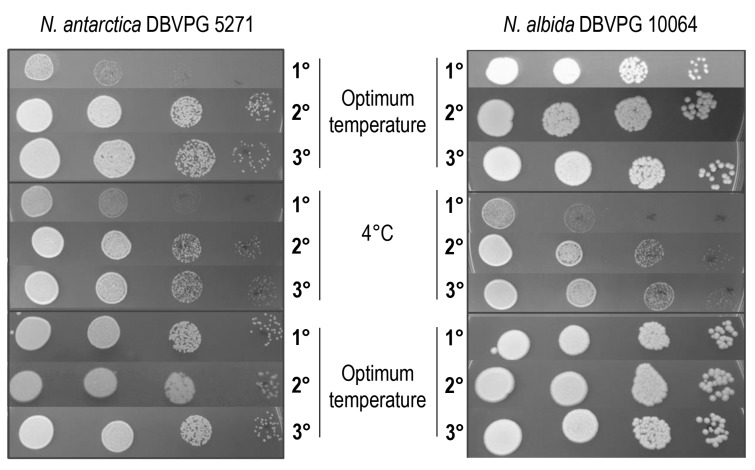
Growth of serial diluted suspensions of *N. antarctica* DBVPG 5271 and *N. albida* DBVPG 10064 during the seeding and incubation cycles. Each suspension was inoculated in Petri dishes containing YEPG agar medium as single spot (10 µL).

This trend was confirmed by the serial diluted suspensions inoculated as single spots in Petri dishes (Figure 3). At the respective optimum temperatures, the growth of *N. albida* strain was appreciably more abundant than *N. antarctica*, but when the strains were moved to 4 °C, the two cultures showed a similar growth. In particular, the observable growth of *N. antarctica* at 4 °C was similar to that observed at optimum incubation temperature while that of *N. albida* was less abundant than that at 25 °C (Figure 3).

### 3.2. Effect of Cold stress on DNA Methylation

**Figure 4:**
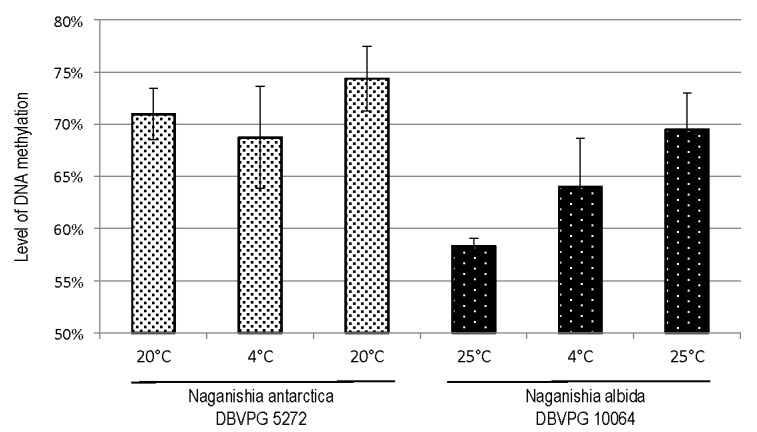
Relative level of DNA methylation as inferred by MSAP in *N. antarctica* DBVPG 5271 and *N. albida* DBVPG 10064 during the seeding and incubation cycles.

After MSAP fingerprint, a total of 550 and 423 clear and reproducible fragments were amplified from *N. antarctica* and *N. albida* strains, respectively (Table S1). The percentage and the trend of relative level of DNA methylation is reported in Table 1 and Figure 4. Under control conditions (20 °C and 25 °C), the average level of DNA methylation of CCGG sequences was 70.97% ± 2.47% in *N. antarctica* and 58.31% ± 0.72% in *N. albida*. When the strains were incubated at low temperature (cold stress), the two Naganishia strains showed an impaired response in terms of DNA methylation: the percentage of total methylated sequence in psychrophilic *N. antarctica* showed no significant decrease (68.73% ± 4.89%, Table S2) and no significant increase (63.99% ± 4.64%, Table S2) in psychrotolerant *N. albida*. Notably, by taking into consideration only stress-induced loci, *N. antarctica* showed a level of demethylation higher than that of methylation (58.01% vs. 41.99%), whereas *N. albida* had an opposite behavior with a percentage of demethylated loci lower than that of methylated ones (41.27% vs. 58.78 %). After recovery from cold stress condition the psychrophilic *N. antarctica* showed a level of methylation (74.36% ± 3.10%) and a banding pattern close to those observed under control conditions. In fact, the changes in DNA methylation between T3 and T1 were not statistically significant. On the contrary, the methylation of psychrotolerant *N. albida* continued to a level that was significantly different (*p* < 0.001) from that of control conditions (69.50% ± 3.48% vs. 58.31 ± 0.72, Table S2).

Consistent with the approach used by Karan et al. [48], all possible banding patterns between control and cold stress condition in *N. antarctica* and *N. albida* strains were compared to identify the changes in cytosine methylation patterns. Sixteen banding patterns were apparent from the MSAP analysis (Table 2). Patterns A–D represent monomorphic classes in which the methylation pattern is the same following either the control or the cold stress condition. Patterns E–J are indicative of cytosine demethylation, whereas possible cytosine methylation events induced by cold stress are represented by patterns K–P.

Out of 550 and 423 fragments, 70.8% and 56.2% of CCGG sites remained unchanged after the imposition of cold temperature to psychrophilic *N. antarctica* and psychrotolerant *N. albida* strains, respectively (Table 2). Under experimental temperature (4 °C) conditions, the percentages of demethylated sites were 17.63% and 18.12%, whereas the percentages of methylated sites were 11.5% and 25.7% in *N. antarctica* and *N. albida*, respectively (Table 2). This indicates more DNA methylation events in cold stressed psychrotolerant than in cold stressed psychrophilic strain (Table 2).

**Figure 5:**
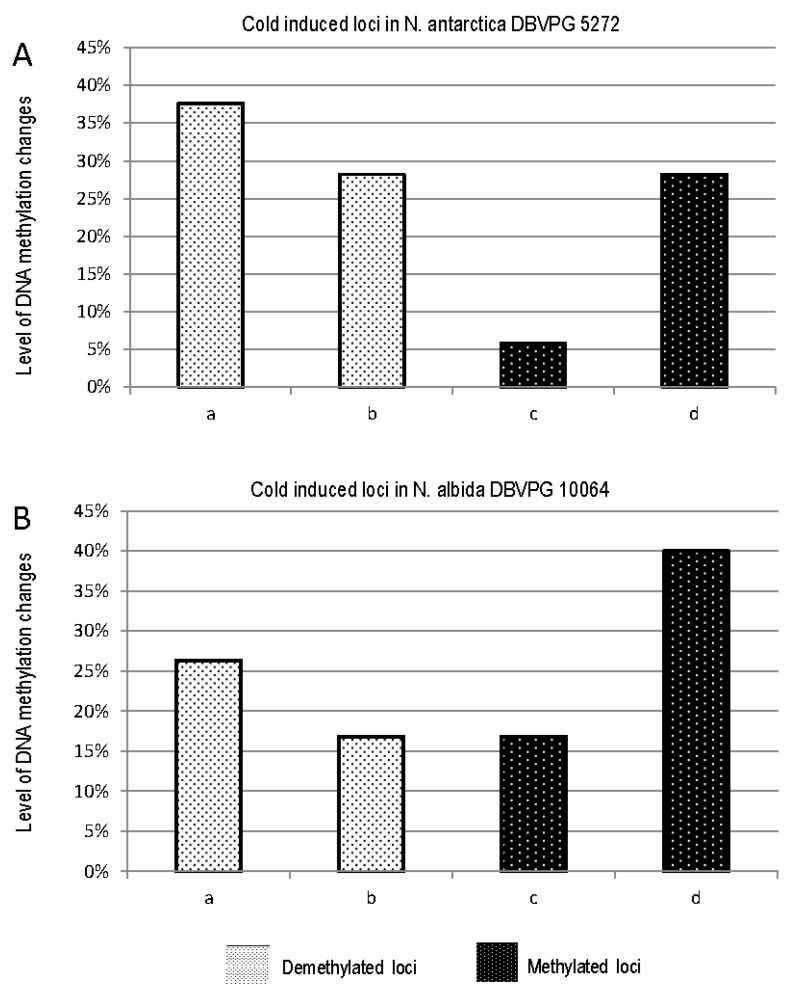
Demethylated and methylated loci of *N. antarctica* DBVPG 5271 (**A**) and *N. albida* DBVPG 10064 (**B**) induced by cold stress and changed (a,c) or unchanged (b,d) after recovery as in Table 2.

To identify the DNA methylation changes (i.e., demethylation or methylation under experimental conditions and subsequent recovery), all differentially methylated DNA fragments have been clustered into seven classes. As indicated in Table 3, the **a**–**c** classes included fragments with DNA demethylation induced by cold stress, the **d**–**f** classes comprised methylated DNA fragments induced by cold stress, and the **g** and **h** classes included DNA fragments for which cold stress had no effect on their methylation status. Interestingly class d, representing the number of DNA patterns methylated by cold stress that remained methylated after recovery, is higher for *N. albida* than for *N. antarctica* (38 vs. 24, Table 3). In particular, after recovery, both strains showed a number of cold stress demethylated loci that were re-methylated (returning to their original status) higher than the unchanged one (a and b classes, respectively; Figure 5). Conversely, the number of cold stressed methylated loci changed after recovery (c class, Figure 5) was lower than that of unchanged loci (d class, Figure 5) in both strains.

### 3.3. Sequencing of Differentially Methylated DNA Fragments

A total of 12 polymorphic fragments were selected, cloned, and sequenced. Four sequences could not be used for BLASTX analysis because they were shorter (55 to 80 bp) than the set limit (100 bp). The sequence length of the residual eight fragments ranged between 116 bp and 287 bp. Two fragments, B1 and B3 (Table 4), did not match to any known sequences of NCBI database (no similarity found). Five other sequences showed a high degree of similarity with those belonging to yeasts (*Cryptococcus amylolentus*, *Cryptococcus neoformans* var. *grubii*, and *S. cerevisiae*) and one with sequence of a cyanobacterium genus (*Cyanothece* sp.) (Table 4). Fragments B2 and B7 were particularly interesting; in fact, they were significantly associated to a gene encoding for elongation factor G of *Cr. neoformans* (OWZ31767). Analogously B6 and B4 showed high similarity with the chitin synthase export chaperon of *Cr. amylolentus* (XM019140565). 

## 4. Discussion

Earth cold environments (i.e., deep seas, cold deserts, and glacial habitats) are known to be colonized by a large microbial diversity, including yeasts, which have developed specific physiological adaptations to increase their chances to survive in such harsh conditions [10]. Some authors demonstrated that laboratory-simulated cold or freeze–thaw conditions may selectively increase the abundance of specific yeast species. These studies were carried out using two strains of *Goffeauzyma gilvescens* (former *Cryptococcus gilvescens*) and *Cryptococcus* sp., which were known to adapt well and duplicate under several stressing conditions such as low nutrients, reduced water availability, low temperature, thermal fluctuations, high UV irradiation. Moreover, after a few cycles, these species became dominant [45,49]. 

Therefore, the above cold-adapted yeasts can survive or even grow at very low temperatures thanks to their activity of decomposition of organic macromolecules through cold-adapted enzyme secretion, thus playing a key role in the carbon cycle [20,45,50,51]. Moreover, they have evolved additional adaptation strategies such as reduction of growth rates, increase of membrane fluidity (changing the composition of fatty acids), synthesis of protecting proteins and cryoprotectant macromolecules (glycerol and trehalose), and other changes at molecular, physiological and metabolic level [6].

In this context, the present work was aimed at investigating the role of DNA methylation in two different yeast species belonging to the same genus, one psychrophilic (*N. antarctica*) and one psychrotolerant (*N. albida*), as a possible response to cold stress. DNA methylation has recently been studied in *Candida albicans*; *Metschnikowia reukaufii*; *Cryptococcus laurentii* (now *Papiliotrema laurentii*); and some species within the Kluyveromyces, Candida, Schizosaccharomyces, and Saccharomyces genera [39,40,42,52]. Despite the involvement of DNA methylation in the rapid response to biotic and abiotic stresses has been widely investigated in plants [46,53,54,55,56,57], none of the above-mentioned studies have taken into consideration the possible involvement of DNA methylation as a possible response induced in yeast species by cold stress. 

As already demonstrated in a few studies [19,58], the growth of psychrophilic yeasts at their optimum temperature was slower than that observed on psychrotolerant ones and this could be considered as an ecological advantage in nutrient-poor environments. This hypothesis is consistent with results herein reported: when the two species were grown at their respective optimum temperature, *N. antarctica* produced a lower quantity of biomass than that produced by *N. albida*, but this difference disappeared when the two strains were grown under cold stress (4 °C). In fact, *N. albida* dry mass decreased consistently, whereas *N. antarctica* increased reaching the same level of biomass for both species. Therefore, incubation at low temperature did not significantly influence the growth of *N. antarctica* while it caused a consistent stress for *N. albida* which, despite its broader range of growing temperature, did not show a prompt adaptation to the incubation at 4 °C. 

The putative role of DNA methylation in the different adaptation of the two strains to cold stress was therefore investigated. The experimental design (Figure 1) was aimed at studying the strains only after a period of stability at the incubation conditions reducing transitional physiological changes. To the best of knowledge, this is the first time that a psychrophilic and a psychrotolerant species are compared in relation to epigenetic response to cold stress. Considering only loci specifically induced by cold stress in both strains, a different behavior is evident. *N. antarctica,* compared to *N. albida*, exhibits apparently a clear genomic strategy in response to cold by demethylating genes, possibly indicating a successful strategy to survive at low temperatures. This hypothesis is apparently confirmed by the observation that only *N. antarctica* strain increases either number of viable cells, amount of biomass, or dry weight during stress revealing a distinctive aptitude to efficiently live and proliferate at low temperature. Moreover, when the overall changes in DNA methylation are considered, the number of methylated loci in *N. antarctica* strain was almost stable in the three steps and this pattern does not occur stochastically in stressed and not stressed cells but seems to be controlled by a stable epigenetic program associated to cold adaptation. On the other hand, it is possible to note a continuous increase of relative level of DNA methylation in *N. albida* (Figure 4) that could resemble an unpaired response after cold.

The different expression of yeast genes involved in transcription and translation in relation to the decrease in temperature, has received the attention of some authors. The cold response of the model organism *S. cerevisiae* using transcriptomic approaches is described and reviewed in Sahara et al. [23] and Aguilera et al. [28]. Tronchoni et al. [24] compared the cold stress response of *S. cerevisiae* (mesophilic species) and *Saccharomyces kudriavzevii* (psychrotolerant species) using transcriptomic and functional approaches, whereas Wong et al. [29] analyzed the cold-adaptation strategies of *G. antarctica* PI12. On the other hands, Tsuji [20] focused for the first time the attention to a psychrophilic yeast species (the Antarctic basidiomycetous yeast *M. blollopis*). 

In the present study, the sequences of differentially methylated DNA fragments resulted to be associated to two main genes: elongation factor G and chitin synthase export chaperon. Elongation factors are proteins which facilitate protein translational and mitochondrial efficiency. The alterations in the expression of these genes are in accordance with the previous literature that associates cold shock events to the activation of genes related to the translation machinery as a way to compensate an initial decrease or inefficient synthesis of protein that follows the cold stress [23,24,27,28]. 

Chitin is one of the minor components of yeasts cell wall in and is normally deposited as a ring in the neck between the mother cell and the emerging bud. In *S. cerevisiae* mutants, it was demonstrated that delocalized chitin in lateral walls can increase to as much as 20% of the wall components, as cell wall stress response (e.g., cells characterized by a deficit of other wall components as b-glucan, mannan, O-linked glycans, and GPI anchors) [59]. In *Cryptococcus neoformans* mutants, the defect in the palmitoylation and localization of a few target genes, including Chs3 chitin synthase, reduced their viability when subjected to antifungal activity stress (caspofungin tolerance) [60]. The alteration of the synthesis of chitin could therefore be associated to an increase or a reduction of cell duplication as well as to the lack of wall components due to reduction of proteins/enzymes synthesis. Both events can be associated to cold stress responses and can justify the methylation changes that could cause activation/inactivation of these genes.

In conclusion, DNA methylation has so far been poorly explored in yeast species, even if it represents an ideal approach to evaluate the responses of different individuals to different kinds of stresses. Our work demonstrated an involvement of DNA methylation in the response of psychrophilic and psychrotolerant yeast strains to cold stress. Based on these positive results we are planning a genome-wide investigation of DNA methylation in these and other species under several stresses.

## Figures and Tables

**Table 1:** DNA methylation changes of *N. antarctica* DBVPG 5271 and *N. albida* DBVPG 10064 at different temperatures: optimum and cold stress.

	*N. antarctica* DBVPG 5271								
	20 °C			4 °C			20 °C		
MSAP Band Type	5271-1	5271-2	5271-3	5271-1	5271-2	5271-3	5271-1	5271-2	5271-3
I	175	155	149	164	202	150	157	143	123
II	45	120	71	66	97	63	70	78	89
III	55	31	50	43	58	89	61	67	55
IV	275	244	280	277	193	248	262	262	283
Tot. Amplified bands	550	550	550	550	550	550	550	550	550
Tot. methylated bands	375	395	401	386	348	400	393	407	427
Fully methylated bands	330	275	330	320	251	337	323	329	338
MSAP (%) ^a^	68.18	71.82	72.91	70.18	63.27	72.73	71.45	74.00	77.645
Fully methylated ratio (%) ^b^	60.00	50.003	60.00	58.18	45.64	61.27	58.73	59.82	61.45
Hemimethylated ratio (%) ^c^	8.18	21.82	12.91	12.00	17.64	11.45	12.73	14.18	16.18
Mean MSAP (%)	70.97			68.73			74.36		
	***N. albida* DBVPG 10064**								
	**25 °C**			**4 °C**			**25 °C**		
**MSAP Band Type**	**10064-1**	**10064-2**	**10064-3**	**10064-1**	**10064-2**	**10064-3**	**10064-1**	**10064-2**	**10064-3**
I	177	173	179	140	142	175	112	137	138
II	82	108	77	94	121	79	78	87	57
III	50	46	77	45	58	54	99	70	104
134	114	96	90	144	102	115		129	124
Tot. Amplified bands	423	423	423	423	423	423	423	423	423
Tot. methylated bands	246	250	244	283	281	248	311	286	285
Fully methylated bands	164	142	167	189	160	169	233	199	228
MSAP (%) ^a^	58.16	59.10	57.68	66.90	66.43	58.63	75.52	67.61	67.38
Fully methylated ratio (%) ^b^	38.77	33.57	39.48	44.68	37.83	39.95	55.08	47.04	53.90
Hemimethylated ratio (%) ^c^	19.39	25.53	18.20	22.22	28.61	18.68	18.449	20.57	13.48
Mean MSAP (%)	58.31			63.99			69.50		

^a^ MSAP (%) = [(II + III + IV)/(I + II + III + IV)] × 100; ^b^ Fully methylated ratio (%) = [(III + IV)/(I + II + III + IV)] × 100; ^c^ Hemimethylated bands (%) = [(II)/(I + II + III + IV)] × 100. Type I indicated absence of methylation due to the presence of bands in both *Eco*RI/*Hpa*II and *Eco*RI/*Msp*I digest; type II bands appeared only in *Eco*RI/*Hpa*II digestion, but not in the *Eco*RI/*Msp*I digest; type III generated bands obtained in *Eco*RI/*Msp*I digest, but not in the *Eco*RI/*Hpa*II digest; and type IV represents the absence of band in both enzyme combinations.

**Table 2:** Analysis of DNA methylation patterns in *N. antarctica* DBVPG 5271 and *N. albida* DBVPG 10064 under cold conditions compared with control conditions.

*N. antarctica* DBVPG 5271								
Pattern	Class	20 °C		4 °C		Rep1	Rep2	Rep3
		*Hpa*II	*Msp*I	*Hpa*II	*Msp*I			
**No change**	A	1	0	1	0	34	44	43
	B	0	1	0	1	23	14	23
	C	1	1	1	1	146	118	112
	D	0	0	0	0	242	144	225
	**Total**					**445 (80.9%)**	**320 (58.2%)**	**403 (73.3%)**
**Demethylation**	E	1	0	1	1	1	47	12
	F	0	1	1	1	13	5	20
	G	0	0	1	1	4	32	6
	H	0	1	1	0	3	1	1
	I	0	0	1	0	21	47	16
	J	0	0	0	1	8	21	33
	**Total**					**50 (9.1%)**	**153 (27.9%)**	**88 (16.0%)**
**Methylation**	K	1	1	1	0	8	5	3
	L	1	1	0	1	10	19	30
	M	1	1	0	0	11	13	4
	N	1	0	0	1	2	4	3
	O	1	0	0	0	8	25	13
	P	0	1	0	0	16	11	6
	**Total**					**55 (10.0%)**	**77 (14.0%)**	**59 (10.7%)**
***N. albida* DBVPG 10064**								
**Pattern**	**Class**	**25 °C**		**4 °C**		**Rep1**	**Rep2**	**Rep3**
		*Hpa*II	*Msp*I	*Hpa*II	*Msp*I			
**No change**	A	1	0	1	0	42	57	37
	B	0	1	0	1	11	24	20
	C	1	1	1	1	112	116	128
	D	0	0	0	0	77	41	48
	**Total**					**242 (57.2%)**	**238 (56.3%)**	**233 (55.1%)**
**Demethylation**	E	1	0	1	1	8	19	9
	F	0	1	1	1	12	1	27
	G	0	0	1	1	8	6	11
	H	0	1	1	0	4	7	9
	I	0	0	1	0	12	36	22
	J	0	0	0	1	17	13	9
	**Total**					**61 (14.4%)**	**82 (19.4%)**	**87 (20.6%)**
**Methylation**	K	1	1	1	0	36	21	11
	L	1	1	0	1	13	17	23
	M	1	1	0	0	16	19	17
	N	1	0	0	1	4	4	2
	O	1	0	0	0	28	28	29
	P	0	1	0	0	23	14	21
	**Total**					**120 (28.4%)**	**103 (24.3%)**	**103 (24.3%)**

**Table 3:** Number of DNA methylation changes of *N. antarctica* DBVPG 5271 and *N. albida* DBVPG 10064 under cold stress condition with definition of arbitrary classes.

	a	b	c	d	e	f	g
***N. antarctica*** **DBVPG 5271**	32	24	5	24	64	278	123
***N. albida*** **DBVPG 10064**	25	16	16	38	58	140	130

(a) Demethylated by 4 °C incubation but re-methylated after recovery; (b) demethylated by 4 °C incubation, and remaining hypomethylated after recovery; (c) methylated by 4 °C incubation, but demethylated after recovery; (d) methylated by 4 °C incubation, and remaining methylated after recovery; (e) DNA methylation pattern remained unchanged during 4 °C incubation, but changed after recovery; (f) DNA methylation pattern was unchanged during 4 °C incubation, and remained unchanged after recovery; and (g) total DNA methylation patterns that were involved in methylation changes.

**Table 4:** Functional association of the methylated fragments. X = presence of amplification product. The complete scoring of samples is given in Table S1.

Frag	bp	BLASTX Analysis		e-value	Accession Number	Banding Profile								
Code		Similarity	Species			C1	C2	C3	S1	S2	S3	R1	R2	R3
B1	116	No similarity found	----		----	**X**	**X**	**X**				**X**	**X**	**X**
B2	217	Elongation factor G, mitochondrial	*Cryptococcus neoformans* var. *grubii* c45	1 × 10^−14^	OWZ31767	**X**	**X**	**X**				**X**	**X**	**X**
B3	126	No similarity found	----		----	**X**	**X**	**X**				**X**	**X**	**X**
B4	287	Chitin synthase export chaperone	*Cryptococcus amylolentus* CBS 6039	1 × 10^−9^	XM019140565				**X**	**X**	**X**			
B5	143	Low-affinity glucose transporter	*Saccharomyces cerevisiae*	3 × 10^−1^	CAA47735	**X**	**X**	**X**				**X**	**X**	**X**
B6	284	Chitin synthase export chaperone	*Cryptococcus amylolentus* CBS 6039	2 × 10^−11^	XM019140565				**X**	**X**	**X**			
B7	208	Elongation factor G, mitochondrial	*Cryptococcus neoformans* var. *grubii* c45	1 × 10^−3^	OWZ31767				**X**	**X**	**X**			
B8	210	AcrB/AcrD/AcrF family protein	*Cyanothece* sp. ATCC 51142	2 × 10^−15^	CP000806				**X**	**X**	**X**			

## Supplementary Materials

The following are available online at https://www.mdpi.com/2076-2607/8/2/296/s1, Table S1: Scoring dataset MSAP, Table S2: chi square control for the evaluation of the significance of the percentage of DNA methylation of *N. antarctica* DBVPG 5271 and *N. albida* DBVPG 10064 during the seeding and incubation cycles, Table S3: Methylation Sensitive Amplified Polymorphism (MSAP) primers combination.
