# Supplementary material for: DNA Methylation Changes Induced by Cold in Psychrophilic and Psychrotolerant Naganishia Yeast Species
Source: Microorganisms. 2020 Feb 20;8(2):296. doi: 10.3390/microorganisms8020296 (PMC7074839; doi:10.3390/microorganisms8020296)
Supplement: Supplementary file 1 [file microorganisms-08-00296-s001.pdf]

DNA methylation changes induced by cold in psychrophilic and psychrotolerant Naganishia yeast species

Benedetta Turchetti*, Gianpiero Marconi, Ciro Sannino, Pietro Buzzini, Emidio Albertini
Department of Agricultural, Food and Environmental Sciences, University of Perugia, Italy
B. Turchetti and G. Marconi contributed equally to this work.

Table S2 chi square control for the evaluation of the significance of the percentage of DNA

N. antarctica DBVPG 5271							
**Meth pattern**	**treatment (°C)**			**(obs-exp)^2/exp**			
	**T1 (20)**	**T2 (4)**	**T3 (20)**	**T2 vs T1**	**T3 vs T2**	**T3 vs T1**	
**Un-methy**	160	172	141	0.65	4.29	1.59	chi square
**Methylated**	390	378	409				
**total**	550	550	550				
				ns	< 0.05	ns	p-value
				1	1	1	DF

N. albida DBVPG 10064							
**Meth pattern**	**treatment (°C)**			**(obs-exp)^2/exp**			
	**T1 (25)**	**T2 (4)**	**T3 (25)**	**T2 vs T1**	**T3 vs T2**	**T3 vs T1**	
**Un-methy**	176	152	129	2.87	2.90	11.48	chi square
**Methylated**	247	271	294				
	423	423	423				
			P-value	ns	ns	< 0.001	p-value
			DF	1	1	1	DF

Table S3 Methylation Sensitive Amplified Polymorphism (MSAP) primers combination

**Adapter and Primer**	***EcoR* I (5'-3')**	***Hpa* II / *Msp* I (5'-3')**
Adapter 1	CTCGTAGACTGCGTACC	GATCATGAGTCCTGCT
Adapter 2	AATTGGTACGCAGTCTAC	CGAGCAGGACTCATGA
Pre-amplification primers	GACTGCGTACCAATTC+C	GATGAGTCTAGAACGG+A
Selective amplification primers	E+CCA<br>E+CAA<br>E+CAC<br>E+CAG	HM+AAT<br>HM+ACT<br>HM+ATC

In the selective amplification, every E+NNN was used in combination with every HM+NNN primer for a total of 12 combinations

Table S1 Scoring dataset MSAP

		Hpa II								
		(C = 25°C)				(S = 4°C)				
	PM	C1	C2	C3	sum	S1	S2	S3	sum	R1
**Comb_1**	51	0	0	0	0	0	1	1	2	1
	55	0	0	1	1	0	0	0	0	0
	58	1	1	1	3	1	1	1	3	1
	65	0	0	0	0	0	0	0	0	0
	72	1	1	1	3	1	1	1	3	0
	76	1	1	0	2	1	1	1	3	1
	80	1	1	1	3	1	1	1	3	1
	88	0	0	0	0	0	0	0	0	0
	89	1	1	1	3	1	1	1	3	1
	91	1	1	0	2	1	1	1	3	1
	93	0	0	0	0	0	1	0	1	0
	108	1	0	0	1	1	1	1	3	1
	111	0	0	0	0	0	0	0	0	0
**B1**	**116**	1	1	1	3	1	1	1	3	1
	119	1	1	1	3	1	1	1	3	1
	126	1	1	1	3	1	1	1	3	1
	140	1	1	0	2	1	1	1	3	1
	143	0	0	0	0	0	1	0	1	0
	146	1	1	1	3	1	1	1	3	0
	150	0	0	1	1	0	1	0	1	0
	167	1	1	1	3	1	1	1	3	1
	168	1	1	1	3	1	1	1	3	0
	170	0	0	0	0	1	0	0	1	0
	171	0	0	0	0	0	0	0	0	0
	179	0	0	0	0	0	0	0	0	0
	180	1	1	1	3	1	1	1	3	0
	181	0	0	0	0	0	1	0	1	0
	202	1	1	1	3	1	1	1	3	0
	203	1	1	1	3	1	1	1	3	1
	206	0	0	0	0	1	0	0	1	0
	207	1	0	0	1	1	1	1	3	1
	211	1	1	1	3	1	1	1	3	1
**B2**	**217**	1	1	1	3	1	1	1	3	1
	227	1	1	1	3	1	1	1	3	1
	230	0	0	0	0	0	0	1	1	0
	247	0	0	0	0	0	1	1	2	0
	251	0	0	0	0	0	1	0	1	0
	156	1	1	1	3	1	1	1	3	0
	275	0	1	1	2	0	1	0	1	1
	276	1	1	1	3	1	1	1	3	1
	281	1	1	0	2	0	1	1	2	1
	303	1	1	1	3	1	1	1	3	1
	312	1	1	0	2	0	0	0	0	0
	318	1	1	1	3	1	1	1	3	1
	336	1	1	1	3	1	1	1	3	1
	377	1	1	1	3	1	1	1	3	1
	385	0	0	1	1	0	0	0	0	0
	423	1	1	1	3	1	1	1	3	1
	438	1	1	1	3	1	1	1	3	1
**Comb_2**	51	0	0	0	0	0	1	1	2	0

	55	1	0	0	1	1	1	1	3	0
	59	1	1	1	3	1	1	1	3	0
	63	0	0	0	0	0	0	0	0	0
	72	0	1	1	2	1	1	1	3	0
	77	1	1	1	3	1	1	1	3	0
	80	1	1	1	3	1	1	1	3	0
	82	0	0	0	0	0	0	0	0	0
	87	0	0	0	0	0	0	0	0	0
	88	1	1	0	2	1	1	1	3	0
	89	1	1	1	3	1	1	1	3	0
	90	0	0	0	0	0	0	0	0	0
	92	0	0	0	0	0	0	0	0	0
	98	0	0	0	0	0	1	1	2	0
	110	1	0	0	1	0	1	1	2	0
		1	1	1	3	1	1	1	3	1
	135	0	0	0	0	0	0	0	0	0
	136	0	0	0	0	0	0	0	0	0
	138	1	1	1	3	1	1	1	3	0
	140	1	1	1	3	1	1	1	3	0
	149	1	1	1	3	0	0	0	0	0
	150	1	1	1	3	1	1	1	3	0
	158	1	1	1	3	1	1	1	3	0
	164	1	1		2	1	1	1	3	0
	170	1	1	1	3	1	1	1	3	0
	179	0	0	0	0	0	0	0	0	0
	180	1	1	1	3	1	1	1	3	0
	194	1	1	1	3	1	1	1	3	0
	202	0	0	0	0	0	1	1	2	0
	218	0	0	0	0	0	0	0	0	0
	221	0	0	0	0	0	1	1	2	0
	227	1	1	0	2	0	0	0	0	0
	228	1	1	1	3	0	0	0	0	0
	265	1	1	1	3	1	1	1	3	0
	266	1	1	1	3	1	1	1	3	0
	385	0	1	0	1	0	0	0	0	0
	423	1	1	1	3	1	1	1	3	0
	469	1	0	0	1	1	0	0	1	0
**Comb_3**	53	1	1	1	3	1	1	1	3	1
	60	1	1	1	3	1	1	1	3	1
	64	0	0	1	1	0	0	0	0	0
	65	1	1	1	3	1	1	1	3	1
	70	0	0	0	0	0	0	0	0	0
	79	1	1	1	3	1	1	1	3	1
	92	0	0	1	1	0	0	0	0	0
	97	1	1	0	2	1	1	1	3	1
	100	1	1	1	3	1	1	1	3	1
	104	0	0	0	0	0	0	0	0	0
	127	1	0	1	2	0	0	0	0	0
	131	1	1	0	2	1	0	0	1	1
	132	1	0	1	2	1	0	0	1	0
	133	1	1	1	3	1	0	1	2	1
	135	1	0	1	2	1	0	0	1	0
	137	1	1	1	3	1	1	1	3	1
	138	1	1	1	3	1	1	1	3	1
	140	1	1	1	3	1	1	1	3	1
	169	1	1	1	3	1	0	0	1	1
	172	1	1	1	3	1	0	0	1	1
	173	1	1	1	3	1	1	1	3	1
	199	0	0	0	0	0	0	0	0	0

	204	0	1	0	1	0	0	0	0	0
	205	1	1	1	3	1	0	0	1	1
	206	1	1	1	3	1	0	0	1	1
	208	1	1	1	3	1	1	1	3	1
	209	1	1	1	3	1	1	1	3	1
	211	1	1	0	2	1	0	0	1	1
	237	1	1	0	2	0	0	0	0	1
	239	0	0	0	0	1	0	0	1	0
	241	1	1	1	3	1	0	0	1	1
	244	1	1	1	3	1	0	0	1	1
	274	1	1	0	2	1	0	0	1	1
	277	1	1	1	3	1	0	0	1	1
	312	1	1	0	2	0	0	0	0	1
	314	1	1	1	3	0	0	0	0	1
	325	1	1	1	3	0	0	0	0	1
	330	1	1	1	3	1	1	1	3	1
	331	1	1	1	3	1	1	0	2	1
	347	1	1	0	2	0	0	0	0	1
	350	1	1	1	3	0	0	0	0	0
	352	1	1	1	3	1	1	1	3	1
	353	1	1	0	2	1	0	0	1	1
	377	0	1	0	1	0	0	0	0	1
	457	1	1	1	3	1	0	0	1	1
**Comb_4**	52	1	1	1	3	1	1	1	3	1
	60	1	1	1	3	1	1	1	3	1
	65	0	0	0	0	0	0	0	0	0
	69	1	1	1	3	1	0	0	1	1
	74	0	1	1	2	1	0	0	1	1
	75	1	1	1	3	1	1	1	3	1
	77	1	1	1	3	1	1	1	3	1
	95	0	0	0	0	0	0	0	0	0
	97	0	0	0	0	0	1	1	2	0
	101	1	1	1	3	1	1	1	3	1
	106	0	0	0	0	1	0	0	1	0
	107	1	0	0	1	0	1	0	1	0
	107	0	1	1	2	0	0	1	1	1
	110	1	0	0	1	0	0	0	0	0
	130	0	0	0	0	1	0	0	1	0
	152	1	1	1	3	1	1	1	3	0
	157	1	1	1	3	1	1	1	3	0
	173	1	1	1	3	1	1	1	3	1
	176	1	1	1	3	1	1	0	2	1
	186	1	1	1	3	1	1	1	3	1
	188	1	1	1	3	1	1	1	3	1
	211	1	1	1	3	1	1	1	3	1
	212	0	0	0	0	0	1	0	1	0
	213	0	1	1	2	0	1	0	1	0
	234	0	0	0	0	0	0	0	0	0
	247	1	1	1	3	1	1	1	3	1
	266	1	1	1	3	1	1	1	3	1
	277	1	1	1	3	1	1	1	3	1
	283	1	1	1	3	1	1	1	3	1
	299	0	0	0	0	0	1	1	2	0
	301	0	0	0	0	0	1	1	2	0
	310	0	0	0	0	0	0	0	0	0
	347	0	0	0	0	1	1	1	3	0
	348	0	0	0	0	1	1	0	2	0
	374	0	1	1	2	1	1	1	3	1
	375	0	0	0	0	0	1	0	1	0

	378	0	0	0	0	0	1	1	2	0
	407	1	1	1	3	1	1	1	3	1
	453	0	1	0	1	1	1	1	3	0
	454	0	0	0	0	0	1	0	1	0
	455	0	0	0	0	0	1	1	2	0
	485	0	0	0	0	0	1	1	2	0
	486	0	0	0	0	0	1	0	1	0
	488	0	0	0	0	0	1	1	2	0
**Comb_5**	56	1	1	1	3	1	1	1	3	1
	59	0	0	0	0	0	0	0	0	0
	64	0	0	0	0	0	0	0	0	0
	73	0	0	0	0	0	0	0	0	0
	81	0	0	0	0	0	0	0	0	0
	82	1	1	1	3	1	1	1	3	1
	83	0	0	0	0	1	1	1	3	0
	89	0	0	0	0	0	0	0	0	0
	90	0	0	0	0	0	0	0	0	0
	93	0	0	0	0	0	0	0	0	0
	115	1	1	1	3	0	0	0	0	0
	116	0	0	0	0	1	0	0	1	0
	122	0	0	0	0	0	0	0	0	0
	123	0	0	0	0	0	0	0	0	0
	127	0	0	1	1	0	0	0	0	0
	140	0	0	0	0	0	0	0	0	0
	141	1	1	1	3	1	1	1	3	1
	142	1	1	1	3	1	1	1	3	0
	175	1	1	1	3	1	1	1	3	1
	180	1	1	1	3	0	0	0	0	0
	208	1	1	1	3	1	1	1	3	1
	213	1	1	1	3	0	0	0	0	0
	219	0	1	1	2	0	0	0	0	0
	231	1	1	1	3	1	1	1	3	1
	233	1	1	1	3	1	1	1	3	1
	234	1	1	0	2	1	1	0	2	1
	239	1	1	0	2	0	1	1	2	1
	264	1	1	1	3	1	1	1	3	1
	266	1	1	1	3	1	1	1	3	1
	298	1	1	1	3	1	1	1	3	1
	299	1	0	0	1	1	1	0	2	0
	306	1	1	1	3	1	1	1	3	1
	308	1	1	1	3	1	1	1	3	1
	328	1	1	1	3	1	1	1	3	1
	333	1	1	1	3	0	1	1	2	1
	340	1	1	1	3	1	1	1	3	1
	342	1	1	1	3	1	1	1	3	1
	363	1	1	1	3	1	1	1	3	1
	364	0	0	0	0	0	0	0	0	0
	371	1	1	1	3	1	1	1	3	1
	379	0	0	0	0	0	0	0	0	0
	396	1	1	0	2	0	0	1	1	1
	423	1	1	1	3	0	0	0	0	1
	428	1	1	1	3	1	1	1	3	1
	462	0	0	0	0	0	0	0	0	0
	478	1	1	1	3	1	1	1	3	1
**Comb_6**	54	1	1	1	3	1	1	1	3	0
	65	1	1	1	3	1	1	1	3	1
	74	1	1	1	3	1	1	1	3	1
	80	1	1	1	3	1	1	1	3	1
	88	1	1	1	3	1	1	1	3	1

	91	0	0	0	0	0	0	0	0	1
	95	1	1	1	3	1	1	1	3	1
	101	1	1	1	3	1	1	1	3	1
	107	0	0	0	0	0	0	0	0	0
	108	0	0	0	0	0	0	0	0	0
	116	1	1	1	3	0	0	0	0	0
	156	1	1	1	3	0	1	1	2	0
	157	1	1	1	3	0	1	0	1	0
	164	0	0	0	0	0	0	1	1	0
	183	1	1	1	3	1	1	1	3	1
	184	1	1	1	3	1	1	1	3	1
	197	0	0	0	0	1	0	0	1	0
	199	1	1	1	3	0	0	0	0	0
	213	1	1	1	3	1	1	1	3	1
	215	0	1	0	1	0	1	1	2	0
	256	1	1	1	3	0	1	0	1	0
	292	1	1	1	3	0	1	1	2	1
	487	1	1	1	3	1	1	1	3	1
**Comb_7**	55	1	1	1	3	1	1	1	3	1
	63	0	0	0	0	0	1	1	2	1
	72	1	1	1	3	1	0	0	1	1
	78	0	0	1	1	1	0	0	1	0
	82	0	1	1	2	0	1	1	2	1
	84	1	1	1	3	1	1	1	3	1
	87	1	1	0	2	1	1	1	3	1
	93	1	1	1	3	1	1	1	3	1
	100	0	0	1	1	0	0	0	0	0
	102	0	1	0	1	0	1	1	2	1
	104	1	0	0	1	0	0	0	0	0
	108	1	1	1	3	0	0	0	0	0
	112	1	0	1	2	1	0	0	1	0
	117	1	1	1	3	1	0	0	1	1
	118	1	1	1	3	1	1	1	3	1
	119	1	1	1	3	1	1	0	2	1
	137	1	1	1	3	0	0	0	0	0
	146	1	1	1	3	0	0	0	0	0
	152	0	1	0	1	0	1	1	2	1
	153	1	1	1	3	1	1	1	3	1
	154	1	1	1	3	1	1	0	2	1
	174	1	1	1	3	0	0	0	0	1
	176	1	1	1	3	0	0	1	1	1
	184	1	1	1	3	0	0	0	0	0
	185	0	0	0	0	0	0	0	0	1
	186	0	1	0	1	0	0	1	1	1
	200	1	1	1	3	0	0	0	0	1
	202	0	0	0	0	0	0	0	0	0
	208	1	1	1	3	0	1	1	2	1
	217	1	1	0	2	0	0	0	0	0
	219	0	1	1	2	0	0	1	1	1
	240	0	0	0	0	0	0	0	0	0
	241	1	1	0	2	0	1	1	2	1
	272	1	1	1	3	1	1	1	3	1
	275	0	1	0	1	0	0	1	1	1
	342	1	1	1	3	0	0	0	0	1
	361	1	1	1	3	1	1	1	3	1
	388	1	1	1	3	0	0	0	0	1
	429	1	1	0	2	0	0	0	0	1
	449	1	1	1	3	0	0	0	0	1
**Comb_8**	56	0	1	1	2	1	1	1	3	1

	83	1	1	1	3	1	1	1	3	1
	97	1	0	0	1	0	0	0	0	0
	103	1	1	1	3	1	1	1	3	1
	110	1	1	1	3	1	1	1	3	1
	112	0	0	0	0	1	1	1	3	0
	126	1	1	1	3	1	1	1	3	1
	129	0	0	0	0	0	0	0	0	0
	135	0	0	0	0	0	0	0	0	0
	137	1	1	1	3	1	1	1	3	1
	138	0	0	0	0	1	1	1	3	0
	139	0	0	0	0	0	0	0	0	0
	148	0	0	0	0	0	0	0	0	1
	158	0	1	1	2	1	1	1	3	1
	160	0	1	1	2	0	0	0	0	0
	163	0	0	1	1	0	0	0	0	0
	185	0	1	1	2	1	1	1	3	1
	192	0	1	0	1	0	1	1	2	1
	205	0	1	1	2	1	1	0	2	1
	217	0	0	0	0	0	1	1	2	0
	229	1	1	1	3	1	1	1	3	1
	252	0	1	1	2	1	1	1	3	1
	253	0	0	0	0	1	1	1	3	0
	268	0	0	0	0	0	0	0	0	0
	284	1	1	1	3	1	1	1	3	1
	289	1	1	1	3	1	1	1	3	1
	312	0	0	0	0	0	1	1	2	0
	345	1	1	1	3	1	1	1	3	1
	393	0	1	1	2	1	1	1	3	1
**Comb_9**	53	0	0	0	0	0	0	0	0	1
	64	1	1	1	3	1	1	1	3	1
	79	1	1	1	3	1	1	1	3	1
	84	0	0	0	0	0	0	0	0	1
	85	1	1	1	3	1	1	1	3	1
	86	1	1	1	3	1	1	1	3	1
	133	1	1	1	3	0	1	1	2	0
	145	1	1	1	3	1	1	1	3	0
	155	1	1	1	3	1	1	1	3	0
	163	1	1	1	3	1	1	1	3	1
	167	1	1	1	3	1	1	1	3	0
	192	1	1	1	3	1	1	1	3	0
	303	1	1	1	3	1	1	1	3	0
	390	0	0	0	0	0	0	1	1	0
	498	1	1	1	3	1	0	1	2	0
	500	0	0	0	0	0	0	0	0	0
**Comb_10**	50	1	1	1	3	1	1	1	3	0
	52	0	1	1	2	1	1	1	3	0
	54	1	1	1	3	1	1	1	3	0
	77	0	1	1	2	1	1	1	3	0
	78	0	0	1	1	0	0	0	0	0
	80	1	1	1	3	1	1	1	3	0
	83	0	0	0	0	0	0	0	0	0
	84	0	1	1	2	1	1	1	3	0
	90	0	0	0	0	0	0	0	0	0
	96	0	1	1	2	1	1	0	2	0
	100	1	1	1	3	1	1	1	3	0
	106	1	1	1	3	1	1	1	3	0
	107	1	1	0	2	1	1	1	3	0
	108	1	1	1	3	1	1	1	3	0
	109	1	1	0	2	1	1	1	3	0

	110	1	0	0	1	0	1	1	2	0
	111	1	1	0	2	0	1	1	2	0
	114	1	0	0	1	0	1	1	2	0
	116	1	0	0	1	0	1	1	2	0
	133	0	0	0	0	0	0	0	0	0
	141	0	1	1	2	1	1	1	3	0
	142	1	1	1	3	1	1	1	3	0
**B5**	**143**	1	1	1	3	0	0	0	0	1
	163	0	0	1	1	0	0	0	0	0
	175	0	1	0	1	0	1	1	2	0
	176	0	1	0	1	1	1	1	3	0
	178	0	0	0	0	1	1	1	3	0
	192	0	0	0	0	0	1	1	2	0
**B8**	**210**	0	0	0	0	1	1	1	3	0
	218	1	1	1	3	1	1	1	3	0
	229	1	1	1	3	0	0	0	0	0
	250	1	1	1	3	1	1	1	3	0
	251	1	1	1	3	1	1	1	3	0
	254	0	0	0	0	0	1	1	2	0
	281	1	1	1	3	1	1	1	3	0
**B6**	**284**	0	0	0	0	1	1	1	3	0
	302	1	1	1	3	0	0	0	0	0
	314	0	1	1	2	0	0	0	0	0
	353	0	1	1	2	1	1	1	3	0
	354	1	1	1	3	1	1	1	3	0
	362	0	1	1	2	1	1	1	3	0
	380	1	1	1	3	0	0	0	0	0
	381	1	1	1	3	1	1	1	3	0
**Comb_11**	62	1	1	0	2	0	0	0	0	1
	78	0	0	0	0	0	0	0	0	0
	83	1	1	1	3	1	1	1	3	1
	89	1	1	1	3	1	1	1	3	1
	108	1	1	1	3	1	1	1	3	1
	110	1	1	1	3	1	1	1	3	1
	137	1	1	1	3	1	1	1	3	1
	142	1	1	1	3	1	1	1	3	1
	143	1	1	1	3	0	1	1	2	0
	149	1	1	1	3	1	1	1	3	0
	161	1	1	1	3	1	1	1	3	1
	162	1	1	1	3	1	1	1	3	1
	163	1	1	1	3	1	1	1	3	0
	218	1	1	0	2	1	1	1	3	0
	219	0	0	1	1	0	0	0	0	1
	275	1	1	0	2	1	1	1	3	0
	278	0	0	1	1	0	0	0	0	1
	282	1	1	0	2	0	0	1	1	0
	285	0	0	1	1	0	0	0	0	1
	289	1	1	0	2	1	0	1	2	0
	292	0	0	1	1	0	0	0	0	1
	297	1	1	0	2	1	1	1	3	0
	300	0	0	1	1	0	0	0	0	1
	334	0	0	1	1	0	0	0	0	1
**Comb_12**	55	0	0	0	0	0	0	0	0	0
	57	0	0	0	0	0	1	0	1	0
	63	0	0	0	0	0	0	0	0	0
	64	0	0	0	0	0	1	1	2	0
	77	0	0	0	0	0	1	1	2	0
	90	1	1	1	3	0	1	1	2	0
	105	0	0	0	0	0	1	0	1	0

	116	0	0	0	0	0	0	0	0	0
	142	0	1	1	2	0	0	1	1	0
	143	0	0	0	0	0	0	0	0	0
	167	1	1	1	3	0	1	1	2	0
	192	1	1	1	3	0	1	1	2	0
	194	1	1	0	2	1	1	1	3	1
**B7**	**208**	0	0	0	0	1	1	1	3	0
	210	1	1	1	3	1	1	1	3	1
	234	1	1	1	3	1	1	1	3	1
	252	1	1	1	3	0	0	0	0	0
	272	1	1	1	3	1	1	1	3	0
	273	1	1	1	3	1	1	1	3	1
	281	0	1	1	2	0	0	1	1	0
	292	0	0	0	0	0	0	0	0	0
	302	0	0	0	0	0	0	0	0	0
	303	0	1	0	1	0	1	1	2	0
	317	1	1	1	3	1	1	1	3	1
	371	1	1	1	3	1	1	1	3	0
	458	1	1	1	3	0	0	1	1	0

		Hpa II								
		(C = 20°C)				(S = 4°C)				
	PM	C1	C2	C3	sum	S1	S2	S3	sum	R1
**Comb_1**	51	1	1	1	3	1	0	1	2	1
	55	1	0	1	2	1	0	1	2	0
	55	0	0	0	0	0	1	0	1	0
	56	1	0	1	2	1	0	1	2	0
	58	1	1	0	2	0	1	1	2	0
	64	0	0	1	1	1	0	0	1	1
	65	1	1	1	3	1	0	1	2	1
	72	0	1	0	1	0	1	0	1	0
	76	1	1	1	3	1	0	1	2	1
	80	0	1	0	1	0	1	0	1	0
	83	1	1	1	3	1	1	1	3	1
	85	1	0	1	2	0	0	1	1	0
	87	0	0	0	0	0	0	0	0	0
	88	1	0	0	1	1	0	1	2	0
	89	1	1	1	3	1	1	1	3	1
	91	1	1	1	3	1	1	1	3	1
	93	0	0	0	0	0	0	0	0	0
	99	1	0	1	2	1	0	1	2	0
	111	1	1	1	3	1	1	1	3	1
	116	1	1	1	3	0	0	0	0	0
	119	0	1	0	1	0	1	0	1	0
	126	0	1	0	1	0	1	0	1	0
	140	1	1	1	3	1	0	1	2	1
	143	1	1	1	3	1	0	1	2	1
	146	0	0	0	0	0	1	0	1	0
	150	1	1	1	3	1	1	1	3	1
	167	0	1	0	1	0	1	0	1	0
	170	1	1	1	3	1	1	1	3	1
	171	0	0	0	0	0	0	0	0	0
	180	0	1	0	1	0	1	0	1	0
	181	0	0	0	0	0	0	0	0	0
	184	1	1	1	3	1	0	1	2	1
	186	1	0	0	1	0	0	1	1	0

	202	0	0	0	0	0	1	0	1	0
	203	0	1	0	1	0	1	0	1	0
	206	1	0	1	2	0	0	1	1	1
	206	1	1	1	3	1	0	1	2	1
	207	0	1	1	2	0	0	0	0	1
	211	0	1	0	1	0	1	0	1	0
	217	0	1	0	1	0	1	0	1	0
	222	0	0	1	1	1	0	1	2	1
	227	0	1	0	1	0	1	0	1	0
	230	1	1	1	3	1	1	1	3	1
	247	1	1	1	3	1	1	1	3	1
	251	0	0	0	0	0	0	0	0	0
	261	1	1	1	3	1	0	0	1	1
	270	0	0	0	0	0	0	0	0	0
	272	0	0	0	0	0	0	0	0	0
	275	0	1	0	1	0	0	0	0	0
	276	0	1	0	1	0	1	0	1	0
	281	0	1	0	1	1	0	0	1	0
	282	1	1	1	3	1	1	1	3	1
	288	0	0	0	0	1	0	0	1	1
	303	0	1	0	1	0	1	0	1	0
	318	0	1	0	1	0	1	0	1	0
	336	0	1	0	1	0	1	0	1	0
	364	1	0	1	2	1	0	1	2	1
	369	0	0	0	0	1	0	0	1	0
	370	0	0	0	0	1	0	0	1	1
	377	0	1	0	1	0	1	0	1	0
	385	0	0	0	0	0	0	0	0	0
	399	1	1	1	3	1	1	1	3	1
	415	1	0	1	2	0	0	1	1	0
	416	1	1	1	3	1	0	1	2	1
	423	0	1	0	1	0	1	0	1	0
	433	1	1	1	3	1	0	1	2	1
	438	0	1	0	1	0	1	0	1	0
**Comb_2**	51	0	1	1	2	1	1	1	3	1
	55	1	1	1	3	1	1	1	3	1
	59	1	1	1	3	1	1	1	3	1
	62	0	0	0	0	0	0	0	0	0
	63	1	1	1	3	1	1	1	3	1
	65	1	1	1	3	1	1	1	3	1
	72	0	0	0	0	1	1	1	3	1
	75	0	0	0	0	0	0	0	0	0
	77	0	1	1	2	1	1	1	3	1
	80	1	1	1	3	1	1	1	3	0
	82	1	0	0	1	0	0	0	0	1
	87	0	0	0	0	0	0	0	0	0
	88	0	1	1	2	1	1	1	3	0
	89	0	1	1	2	1	1	1	3	0
	92	0	0	0	0	0	0	0	0	0
	100	0	0	0	0	0	0	0	0	0
	113	1	0	0	1	0	0	0	0	1
	116	1	0	0	1	0	0	0	0	1
**B3**	**126**	0	0	0	0	1	1	1	3	0
	136	0	0	0	0	0	0	0	0	0
	138	0	1	1	2	1	1	1	3	0
	140	0	1	1	2	1	1	1	3	1
	149	0	0	0	0	1	1	0	2	0
	150	0	1	1	2	1	1	1	3	0
	158	0	1	1	2	1	1	1	3	0

	170	0	1	1	2	1	1	1	3	0
	180	0	1	1	2	1	1	1	3	0
	218	0	0	0	0	1	1	1	3	0
	222	0	0	0	0	0	0	0	0	1
	232	0	0	0	0	0	0	0	0	1
	248	0	0	0	0	0	0	0	0	1
	265	0	1	1	2	1	1	1	3	0
	266	0	1	1	2	1	1	1	3	0
	270	0	0	0	0	0	0	0	0	0
	272	0	0	0	0	0	0	0	0	0
	288	0	0	0	0	0	0	0	0	1
	369	0	0	0	0	0	0	0	0	1
	423	0	1	1	2	1	1	1	3	0
**Comb_3**	53	1	1	1	3	1	1	1	3	1
	60	1	1	1	3	1	1	1	3	1
	64	0	1	0	1	0	0	0	0	0
	65	0	1	0	1	0	1	0	1	0
	70	0	1	0	1	0	0	0	0	0
	79	1	1	1	3	1	1	1	3	1
	89	1	1	1	3	1	0	1	2	1
	92	0	1	0	1	0	0	0	0	0
	97	1	1	1	3	1	1	1	3	1
	100	0	1	0	1	0	1	0	1	1
	104	0	1	0	1	0	0	0	0	0
	107	0	0	1	1	1	0	0	1	0
	122	1	0	0	1	0	0	1	1	0
	127	0	1	0	1	0	0	0	0	0
	129	1	0	0	1	0	0	1	1	1
	130	1	0	1	2	1	0	1	2	1
	131	0	0	1	1	1	0	1	2	1
	132	0	0	0	0	1	0	0	1	0
	133	0	0	1	1	0	1	0	1	0
	135	0	0	0	0	0	0	0	0	0
	137	0	1	0	1	0	1	0	1	0
	138	0	1	0	1	0	1	0	1	0
	140	0	1	0	1	0	1	0	1	0
	160	1	0	1	2	1	0	1	2	1
	166	1	0	0	1	0	0	1	1	1
	169	0	1	0	1	0	1	0	1	0
	172	0	1	1	2	0	1	1	2	0
	173	1	1	1	3	1	1	1	3	1
	174	1	0	0	1	1	0	1	2	1
	175	0	0	0	0	0	0	0	0	1
	199	0	0	0	0	0	0	1	1	1
	201	0	0	0	0	0	0	0	0	0
	204	0	1	0	1	0	1	1	2	0
	205	0	1	0	1	0	1	1	2	1
	206	1	1	0	2	1	0	1	2	1
	208	1	1	1	3	1	1	1	3	1
	209	0	1	0	1	0	1	0	1	0
	211	1	0	1	2	1	1	1	3	1
	233	0	0	0	0	0	0	1	1	1
	237	0	0	0	0	0	1	1	2	0
	239	0	0	0	0	0	0	1	1	1
	241	0	1	0	1	0	1	0	1	0
	244	0	1	0	1	0	1	0	1	0
	252	1	1	1	3	1	0	1	2	1
	266	0	0	0	0	0	0	0	0	1
	271	0	0	0	0	0	0	0	0	1

	274	1	0	0	1	1	1	1	3	1
	274	0	0	1	1	0	1	0	1	1
	277	0	1	0	1	0	1	0	1	0
**B4**	**287**	0	0	0	0	1	1	1	3	0
	300	0	0	0	0	0	0	0	0	1
	308	1	0	0	1	0	0	1	1	1
	312	0	0	0	0	0	1	0	1	0
	314	0	1	0	1	0	1	0	1	0
	323	0	0	0	0	0	0	1	1	1
	330	0	1	0	1	0	1	0	1	0
	331	0	0	0	0	0	1	0	1	0
	342	0	0	0	0	0	0	0	0	1
	347	0	0	0	0	0	1	0	1	0
	350	0	0	0	0	0	1	0	1	0
	352	0	1	0	1	0	1	0	1	0
	354	0	0	0	0	0	0	0	0	1
	372	0	0	0	0	0	0	0	0	1
	377	0	0	0	0	0	1	0	1	0
	387	1	0	0	1	1	0	1	2	1
	421	0	0	0	0	0	0	0	0	1
	454	0	0	0	0	0	0	0	0	1
	461	0	0	0	0	0	0	0	0	1
**Comb_4**	52	1	1	1	3	1	1	1	3	1
	57	1	1	1	3	1	0	1	2	1
	60	1	1	1	3	1	1	1	3	1
	61	1	0	1	2	1	0	1	2	0
	65	0	0	1	1	0	0	0	0	0
	66	0	0	1	1	1	0	0	1	0
	69	0	1	0	1	0	1	0	1	0
	71	0	0	0	0	0	0	0	0	0
	74	0	1	0	1	0	0	0	0	0
	75	0	1	0	1	0	1	0	1	0
	77	1	1	1	3	1	1	1	3	1
	95	0	0	0	0	0	0	1	1	0
	101	0	1	0	1	0	1	0	1	0
	106	0	1	0	1	0	0	0	0	0
	107	1	0	1	2	1	0	1	2	0
	110	0	1	1	2	1	1	1	3	1
	119	1	1	1	3	1	1	1	3	1
	130	1	1	1	3	1	0	1	2	1
	152	0	1	0	1	0	1	0	1	0
	153	1	0	1	2	1	0	1	2	1
	157	0	1	0	1	0	1	0	1	0
	165	0	0	0	0	0	0	0	0	1
	173	1	1	1	3	1	1	1	3	1
	176	0	1	0	1	0	1	0	1	0
	184	1	0	0	1	0	0	1	1	1
	186	1	1	1	3	1	1	1	3	1
	188	0	1	0	1	0	1	0	1	0
	207	1	0	1	2	1	0	1	2	1
	211	0	1	1	2	0	1	0	1	0
	212	1	1	1	3	1	1	1	3	1
	247	0	1	0	1	0	1	0	1	0
	266	0	1	0	1	0	1	0	1	0
	277	0	1	0	1	0	1	0	1	0
	283	0	1	0	1	0	1	0	1	0
	299	0	0	0	0	0	1	0	1	0
	301	1	1	1	3	1	1	1	3	1
	310	0	0	1	1	1	0	1	2	0

	343	0	0	0	0	0	0	0	0	0
	347	0	0	0	0	0	1	0	1	0
	374	1		1	2	1	0	0	1	1
	374	0	0	0	0	0	1	0	1	0
	378	0	0	0	0	0	1	0	1	0
	407	0	1	0	1	0	1	0	1	0
	453	0	0	0	0	0	1	0	1	0
	455	0	0	0	0	0	1	0	1	0
	479	1	0	1	2	1	0	0	1	0
	485	0	0	0	0	0	1	0	1	0
	488	0	0	0	0	0	1	0	1	0
**Comb_5**	51	1	1	1	3	1	0	1	2	1
	56	1	1	1	3	1	1	1	3	1
	59	1	1	1	3	1	1	1	3	0
	64	1	0	1	2	1	0	0	1	0
	64	0	0	0	0	0	0	0	0	0
	65	1	1	1	3	1	0	1	2	1
	67	1	1	1	3	1	1	1	3	1
	73	0	1	0	1	0	0	0	0	0
	76	1	1	1	3	1	1	1	3	1
	81	0	1	0	1	0	0	0	0	0
	82	1	1	1	3	1	1	1	3	1
	83	1	1	1	3	1	1	1	3	1
	85	0	0	0	0	0	0	0	0	0
	88	1	1	0	2	0	1	1	2	1
	89	0	0	0	0	0	0	0	0	1
	90	1	1	1	3	1	1	1	3	1
	93	0	0	0	0	0	0	0	0	0
	100	1	0	1	2	1		1	2	0
	115	0	0	0	0	0	0	0	0	0
	116	0	0	0	0	0	1	1	2	0
	117	0	0	0	0	0	1	1	2	1
	121	0	0	0	0	0	1	1	2	1
	122	0	0	0	0	0	0	0	0	0
	123	0	0	0	0	0	0	0	0	0
	127	0	1	0	1	0	1	0	1	0
	140	0	0	0	0	0	0	0	0	0
	141	0	1	0	1	0	1	0	1	0
	180	0	1	0	1	0	1	0	1	0
	201	1	1	1	3	1	1	1	3	1
	213	0	0	0	0	0	0	0	0	0
	219	0	0	0	0	0	0	0	0	0
	231	0	1	0	1	0	1	0	1	0
	234	0	0	0	0	0	0	0	0	0
	264	0	0	0	0	0	1	0	1	0
	290	1	1	1	3	1	1	1	3	1
	298	0	0	0	0	0	1	0	1	0
	306	0	1	0	1	0	1	0	1	0
	328	0	1	0	1	0	1	0	1	0
	342	0	1	0	1	0	1	0	1	0
	363	0	0	0	0	0	1	0	1	0
	366	1	1	1	3	1	1	1	3	1
	379	1	1	1	3	1	1	1	3	1
	411	1	1	1	3	1	1	1	3	1
	423	0	1	0	1	0	1	0	1	0
	425	1	1	1	3	1	1	1	3	1
	428	0	1	0	1	0	1	0	1	0
	478	0	1	0	1	0	1	0	1	0
**Comb_6**	53	1	0	1	2	1	0	0	1	1

	55	1	0	1	2	1	0	1	2	0
	61	1	0	1	2	1	0	1	2	1
	64	1	0	1	2	1	0	0	1	0
	65	0	1	0	1	0	1	1	2	0
	66	1	1	1	3	1	0	1	2	1
	71	0	0	0	0	0	0	0	0	0
	74	0	1	0	1	0	1	0	1	0
	80	1	1	1	3	1	1	1	3	1
	88	0	1	0	1	0	1	0	1	0
	91	0	0	0	0	0	0	0	0	1
	93	1	1	1	3	1	0	1	2	1
	95	0	1	0	1	0	1	0	1	0
	101	1	1	1	3	1	1	1	3	1
	107	1	0	1	2	1	0	1	2	0
	108	0	1	0	1	0	0	0	0	0
	119	1	0	1	2	1	0	1	2	0
	123	1	0	1	2	1	0	1	2	1
	130	1	0	1	2	1	1	1	3	1
	153	1	0	1	2	1	0	1	2	1
	156	0	0	0	0	0	1	0	1	0
	157	0	0	0	0	0	1	0	1	0
	164	1	1	1	3	0	1	1	2	1
	167	1	0	1	2	1	0	1	2	1
	172	1	0	0	1	1	0	0	1	1
	176	1	0	1	2	1	0	1	2	1
	183	0	1	0	1	0	1	0	1	0
	184	0	1	0	1	0	1	0	1	0
	186	1	0	1	2	1	0	1	2	1
	197	1	0	1	2	0	0	0	0	0
	211	1	0	1	2	1	0	1	2	1
	213	0	1	0	1	0	1	0	1	0
	256	0	1	0	1	0	1	0	1	0
	273	0	0	0	0	0	0	0	0	1
	292	0	1	0	1	0	1	0	1	0
	309	1	1	1	3	1	0	1	2	1
	314	1	0	1	2	1	0	1	2	1
	347	1	1	1	3	1	0	1	2	1
	364	1	1	1	3	1	1	1	3	1
	420	1	1	1	3	1	0	1	2	1
	469	1	1	1	3	1	0	1	2	0
	487	0	1	0	1	0	1	0	1	0
**Comb_7**	55	1	1	1	3	1	1	1	3	1
	63	0	0	0	0	0	1	0	1	0
	72	1	1	1	3	1	0	1	2	0
	78	1	1	1	3	1	1	1	3	1
	82	0	0	0	0	0	1	0	1	0
	84	1	1	1	3	1	1	1	3	1
	87	0	0	0	0	0	1	0	1	0
	93	0	1	0	1	0	1	0	1	0
	100	0	1	0	1	0	0	0	0	0
	102	0	0	0	0	0	1	0	1	0
	104	0	0	0	0	0	0	0	0	0
	106	1	1	1	3	1	0	1	2	1
	108	0	1	0	1	0	0	0	0	0
	112	1	0	0	1	1	0	1	2	1
	117	0	1	0	1	0	0	0	0	0
	118	0	1	0	1	0	1	0	1	0
	119	0	0	0	0	0	0	0	0	0
	132	0	0	0	0	0	0	0	0	0

	137	0	0	0	0	0	0	0	0	0
	146	1	1	0	2	0	0	1	1	0
	152	0	0	0	0	0	1	0	1	0
	153	0	1	0	1	0	1	0	1	0
	154	0	0	0	0	0	0	0	0	0
	174	0	1	0	1	0	0	0	0	0
	176	0	1	0	1	0	1	0	1	0
	184	0	1	0	1	0	1	0	1	0
	185	1	1	1	3	1	0	1	2	1
	186	0	0	0	0	0	1	0	1	0
	193	1	0	1	2	1	0	1	2	1
	200	0	1	0	1	0	0	0	0	0
	202	1	1	1	3	1	0	1	2	1
	208	0	1	0	1	0	1	0	1	0
	217	0	0	0	0	0	1	0	1	0
	235	1	1	1	3	1	1	1	3	1
	240	1	0	0	1	0	0	0	0	1
	241	0	0	0	0	0	1	0	1	0
	266	1	1	1	3	1	0	1	2	1
	272	0	1	0	1	0	1	0	1	0
	275	0	0	0	0	0	1	0	1	0
	283	1	1	1	3	1	1	1	3	1
	318	0	0	0	0	0	0	0	0	0
	342	1	1	0	2	0	0	0	0	0
	361	0	1	0	1	0	1	0	1	0
	419	1	0	1	2	1	0	1	2	1
	437	1	1	1	3	1	1	1	3	1
	449	0	0	0	0	0	1	0	1	0
	456	1	1	1	3	1	1	1	3	1
	465	1	1	1	3	1	1	1	3	1
	474	1	1	1	3	1	1	1	3	1
	488	0	0	0	0	0	0	1	1	1
	498	0	0	0	0	0	0	1	1	1
**Comb_8**	56	1	1	1	3	0	1	1	2	0
	83	1	1	1	3	1	1	1	3	1
	89	1	1	1	3	1	0	1	2	1
	96	1	1	1	3	1	1	1	3	1
	97	1	1	1	3	1	1	1	3	1
	103	0	1	1	2	0	1	0	1	0
	105	0	0	0	0	0	0	0	0	0
	110	0	1	0	1	0	1	0	1	0
	112	0	0	0	0	0	1	0	1	0
	124	1	0	1	2	1	0	1	2	0
	126	0	1	0	1	0	1	1	2	0
	129	0	0	0	0	0	0	0	0	0
	137	0	1	0	1	0	1	0	1	0
	138	0	0	0	0	0	1	0	1	0
	158	0	0	0	0	0	1	0	1	0
	163	0	1	1	2	1	0	0	1	1
	176	1	1	1	3	1	1	1	3	1
	185	0	0	0	0	0	1	0	1	0
	205	0	0	0	0	0	0	0	0	0
	229	0	1	0	1	0	1	0	1	0
	252	0	0	0	0	0	1	0	1	0
	264	1	0	1	2	0	0	1	1	1
	276	0	0	0	0	1	1	1	3	0
	284	1	1	1	3	1	1	1	3	1
	289	1	1	1	3	1	1	1	3	1
	323	0	0	1	1	1	0	0	1	1

	345	0	0	0	0	0	1	0	1	0
	347	1	1	1	3	1	1	1	3	1
**Comb_9**	53	1	0	1	2	1	0	0	1	0
	57	1	1	1	3	1	1	1	3	1
	64	0	0	0	0	0	1	0	1	0
	79	1	1	1	3	1	1	1	3	1
	84	1	1	1	3	1	1	1	3	1
	85	1	1	1	3	1	1	1	3	1
	86	0	1	0	1	0	1	0	1	0
	133	0	0	0	0	0	1	0	1	0
	148	0	0	0	0	0	0	0	0	0
	155	0	0	0	0	0	1	0	1	0
	163	0	0	0	0	0	0	1	1	0
	165	1	1	1	3	1	1	1	3	1
	167	0	1	0	1	0	1	0	1	0
	171	1	0	1	2	1	0	1	2	1
	172	1	1	1	3	0	0	1	1	1
	173	1	1	1	3	1	1	1	3	1
	184	1	1	1	3	1	1	1	3	1
	192	0	1	0	1	0	1	0	1	0
	193	1	1	1	3	1	1	1	3	1
	207	1	1	1	3	1	1	1	3	1
	303	0	1	0	1	0	1	0	1	0
	390	1	1	1	3	1	1	1	3	1
	415	1	0	1	2	1	1	1	3	1
	498	0	0	0	0	0	1	0	1	0
	500	1	1	1	3	1	1	1	3	1
**Comb_10**	50	0	1	0	1	0	1	0	1	0
	52	0	1	0	1	0	1	0	1	0
	54	1	1	1	3	1	1	0	2	1
	56	1	0	1	2	1	0	0	1	1
	57	1	0	1	2	1	0	0	1	1
	72	1	0	1	2	1	0	0	1	1
	73	1	0	1	2	1	0	0	1	1
	77	0	0	0	0	0	0	0	0	0
	78	1	1	1	3	1	0	0	1	1
	80	0	0	0	0	0	1	0	1	0
	82	1	0	1	2	1	0	0	1	0
	83	0	1	0	1	0	0	0	0	0
	84	0	1	0	1	0	1	0	1	0
	85	1	0	0	1	0	0	0	0	1
	87	1	0	1	2	1	0	0	1	1
	88	1	0	0	1	1	0	0	1	1
	90	0	1	0	1	1	0	0	1	0
	96	0	1	0	1	0	1	0	1	0
	100	1	1	1	3	1	1	0	2	1
	106	1	1	1	3	1	1	0	2	1
	107	1	0	0	1	1	1	0	2	0
	108	0	1	0	1	1	1	0	2	0
	109	0	0	0	0	0	1	0	1	0
	110	0	0	0	0	0	1	0	1	0
	111	1	0	0	1	0	1	0	1	1
	114	0	0	0	0	0	1	0	1	0
	116	0	0	0	0	0	1	0	1	0
	120	1	0	0	1	0	0	0	0	1
	133	1	0	0	1	0	0	0	0	1
	141	0	0	0	0	1	1	0	2	0
	142	0	1	0	1	0	1	0	1	0
	146	1	0	1	2	1	0	0	1	1

	163	0	0	0	0	0	0	0	0	0
	170	1	0	1	2	1	0	0	1	1
	171	1	0	1	2	1	0	0	1	1
	175	0	0	0	0	0	1	0	1	0
	176	0	0	0	0	0	1	0	1	1
	178	0	0	1	1	0	0	0	0	1
	198	0	0	1	1	1	0	0	1	1
	201	1	0	1	2	1	0	0	1	1
	202	1	0	1	2	1	0	0	1	1
	210	0	0	0	0	0	1	0	1	0
	218	1	1	1	3	1	1	0	2	1
	229	0	1	0	1	0	0	0	0	0
	235	1	0	1	2	1	0	0	1	1
	250	0	1	0	1	1	1	0	2	0
	251	0	1	0	1	0	1	0	1	0
	254	0	0	0	0	0	1	0	1	0
	265	1	0	1	2	1	0	0	1	1
	266	1	0	1	2	1	0	0	1	1
	270	1	0	1	2	1	0	0	1	1
	281	0	1	0	1	0	1	0	1	0
	282	1	0	1	2	1	0	0	1	1
	284	1	1	1	3	0	0	0	0	1
	302	0	0	0	0	0	1	0	1	0
	303	0	0	0	0	0	0	0	0	1
	314	0	0	0	0	0	0	0	0	0
	318	0	0	0	0	0	0	0	0	0
	333	1	1	0	2	1	0	0	1	1
	334	1	0	1	2	1	0	0	1	1
	353	0	1	0	1	1	1	0	2	0
	354	0	1	0	1	0	1	0	1	0
	362	0	0	0	0	0	1	0	1	0
	373	1	1	1	3	1	0	0	1	1
	380	0	1	0	1	0	0	0	0	0
	381	0	1	0	1	0	1	0	1	0
	437	1	0	1	2	1	0	0	1	1
	456	1	0	1	2	1	0	0	1	1
	465	1	0	1	2	1	0	0	1	1
	474	0	0	0	0	0	0	0	0	0
**Comb_11**	53	0	0	0	0	0	0	0	0	0
	62	0	0	0	0	0	0	1	1	0
	64	0	0	0	0	0	0	0	0	0
	78	1	0	0	1	0	0	1	1	0
	83	1	1	1	3	1	1	1	3	1
	88	1	1	1	3	1	1	1	3	1
	89	1	1	1	3	1	1	1	3	1
	100	1	0	1	2	1	0	1	2	0
	108	0	1	0	1	0	1	0	1	0
	110	0	1	0	1	0	1	0	1	0
	117	0	0	0	0	0	0	1	1	0
	137	0	1	0	1	0	1	0	1	0
	142	0	1	0	1	0	1	0	1	0
	143	0	0	0	0	0	1	0	1	0
	161	0	0	0	0	0	1	0	1	0
	162	0	1	0	1	0	1	0	1	0
	163	0	0	0	0	0	1	0	1	0
	176	1	1	1	3	1	1	1	3	1
	218	0	0	0	0	0	1	0	1	0
	270	1	0	1	2	1	0	1	2	1
	275	0	1	0	1	0	1	0	1	0

	278	0	0	0	0	0	0	1	1	0
	282	0	0	0	0	0	1	0	1	0
	297	0	1	0	1	0	1	0	1	0
	300	0	0	0	0	0	0	0	0	0
	332	1	1	1	3	1	1	1	3	0
	343	1	1	1	3	1	1	1	3	0
**Comb_12**	53	0	0	0	0	0	0	0	0	0
	55	0	0	0	0	0	0	0	0	0
	56	0	0	0	0	0	0	0	0	0
	57	1	0	1	2	1	1	1	3	0
	63	0	0	0	0	1	0	0	1	0
	64	0	0	0	0	0	0	0	0	0
	77	1	0	0	1	1	1	1	3	0
	85	1	0	0	1	1	0	1	2	0
	90	0	0	0	0	0	1	0	1	0
	99	1	0	1	2	1	0	1	2	0
	105	0	0	0	0	0	0	0	0	0
	116	0	0	0	0	0	0	0	0	0
	129	1	1	1	3	1	1	1	3	1
	142	0	0	0	0	0	1	0	1	0
	143	0	0	0	0	0	0	0	0	0
	167	0	0	0	0	0	1	0	1	0
	184	1	0	1	2	0	0	1	1	0
	192	0	1	0	1	0	1	0	1	0
	194	0	1	1	2	1	0	0	1	1
	208	1	1	0	2	1	1	1	3	0
	210	1	1	1	3	1	1	1	3	1
	224	1	1	1	3	1	1	1	3	1
	234	0	1	0	1	0	1	0	1	0
	252	0	0	0	0	0	1	0	1	0
	270	1	0	1	2	0	0	1	1	1
	272	0	1	0	1	0	1	0	1	0
	273	0	1	0	1	0	1	0	1	0
	280	0	0	0	0	0	0	0	0	0
	281	0	0	0	0	0	1	0	1	0
	302	0	0	0	0	0	0	0	0	0
	303	0	0	0	0	0	1	0	1	0
	317	0	1	0	1	0	1	0	1	0
	345	0	0	0	0	0	0	0	0	0
	354	0	0	0	0	0	0	0	0	0
	371	0	1	0	1	0	1	0	1	0
	381	1	1	1	3	1	1	1	3	1
	390	0	0	0	0	0	0	0	0	0
	414	1	1	1	3	1	1	1	3	1
	458	0	0	0	0	0	1	0	1	0

Strain 10064

						Msp I				
(R = 25°C)			(C = 25°C)				(S = 4°C)			
R2	R3	sum	C1	C2	C3	sum	S1	S2	S3	sum
0	1	2	0	0	1	1	1	0	1	2
0	0	0	0	0	1	1	0	0	0	0
1	1	3	1	1	1	3	1	1	1	3
0	0	0	0	0	1	1	0	0	0	0
1	1	2	1	1	1	3	0	1	0	1
1	1	3	1	1	1	3	1	1	1	3
1	1	3	1	1	1	3	0	1	0	1
0	0	0	0	1	1	2	0	1	0	1
1	1	3	1	1	1	3	1	1	1	3
1	1	3	1	1	1	3	1	1	1	3
0	0	0	0	0	1	1	0	1	0	1
1	0	2	1	1	1	3	0	0	0	0
0	0	0	0	0	1	1	0	0	0	0
1	1	3	1	1	1	3	0	0	0	0
1	0	2	1	1	1	3	0	0	0	0
1	1	3	1	1	1	3	0	1	0	1
1	1	3	1	1	1	3	1	1	1	3
0	0	0	0	0	1	1	0	1	1	2
1	0	1	1	1	0	2	0	0	0	0
0	1	1	0	0	1	1	0	0	0	0
1	0	2	1	1	0	2	0	1		1
1	0	1	0	1	1	2	0	0	0	0
0	1	1	0	0	1	1	0	0	0	0
0	1	1	0	0	1	1	0	1	0	1
0	1	1	0	0	1	1	0	0	0	0
1	1	2	1	1	1	3	0	1	0	1
0	1	1	0	0	1	1	0	1	0	1
1	0	1	1	1	0	2	0	0	0	0
1	0	2	1	1	1	3	1	1	1	3
0	0	0	0	0	0	0	0	0	0	0
1	0	2	1	1	0	2	1	0	0	1
1	0	2	1	1	1	3	1	1	0	2
1	1	3	1	1	1	3	0	0	0	0
1	1	3	1	1	1	3	1	1	1	3
0	0	0	0	0	1	1	0	0	0	0
0	0	0	0	0	0	0	1	0	0	1
0	0	0	0	0	0	0	0	1	1	2
1	0	1	1	1	0	2	0	0	0	0
1	0	2	1	0	1	2	0	0	1	1
1	0	2	1	1	1	3	1	1	1	3
1	0	2	1	1	0	2	1	0	0	1
1	0	2	1	1	0	2	1	1	0	2
0	0	0	0	1	0	1	0	1	0	1
1	1	3	0	0	0	0	0	0	0	0
1	1	3	1	1	1	3	1	1	1	3
1	0	2	0	0	0	0	0	0	0	0
0	1	1	1	0	1	2	0	1	0	1
1	1	3	1	1	1	3	1	1	1	3
1	0	2	0	0	0	0	0	0	0	0
0	0	0	0	0	0	0	0	0	0	0

0	0	0	1	1	1	3	1	1	1	3
1	1	2	1	1	1	3	1	1	1	3
0	0	0	0	0	1	1	0	0	0	0
0	0	0	0	0	1	1	1	1	1	3
1	1	2	1	1	1	3	1	1	1	3
1	1	2	1	1	1	3	1	1	1	3
0	0	0	0	0	1	1	0	0	0	0
0	0	0	1	1	1	3	0	1	1	2
1	0	1	1	1	1	3	1	1	1	3
1	1	2	1	1	1	3	1	1	1	3
0	0	0	0	0	0	0	0	0	0	0
0	0	0	0	1	1	2	0	1	1	2
0	0	0	0	0	0	0	0	0	0	0
1	0	1	0	0	0	0	0	0	0	0
1	1	3	1	1	1	3	1	1	1	3
0	0	0	1	1	1	3	1	1	1	3
0	0	0	1	1	1	3	1	1	1	3
1	1	2	0	0	0	0	0	0	0	0
1	1	2	1	1	0	2	1	1	1	3
0	1	1	1	1	1	3	0	1	1	2
1	1	2	1	1	1	3	1	1	1	3
1	1	2	1	1	1	3	1	1	1	3
1	1	2	0	0	0	0	0	0	0	0
1	1	2	1	1	1	3	1	1	1	3
0	0	0	1	1	1	3		1	1	2
1	1	2	1	1	1	3	1	1	1	3
1	1	2	1	1	1	3	1	1	1	3
0	0	0	0	0	0	0	0	0	0	0
0	0	0	1	0	1	2	0	1	0	1
0	0	0	0	0	1	1	0	0	0	0
1	1	2	0	1	1	2	0	0	0	0
1	1	2	0	0	0	0	0	0	0	0
1	1	2	1	1	1	3	0	0	0	0
1	1	2	1	1	1	3	0	1	1	2
0	0	0	1	1	1	3	0	0	0	0
1	1	2	1	1	1	3	1	1	1	3
1	0	1	0	0	0	0	0	0	0	0
1	1	3	1	1	1	3	1	1	1	3
1	1	3	1	1	1	3	1	1	1	3
0	1	1	0	1	0	1	0	1	0	1
1	1	3	1	1	1	3	1	1	1	3
0	1	1	0	1	0	1	0	1	0	1
1	1	3	1	1	1	3	1	1	1	3
0	1	1	0	1	0	1	0	1	0	1
1	0	2	1	0	1	2	1	0	1	2
1	1	3	1	1	1	3	1	1	1	3
0	1	1	0	1	0	1	0	1	0	1
0	1	1	0	1	0	1	0	1	0	1
0	0	1	1	1	0	2	0	0	1	1
0	0	0	0	1	0	1	0	0	0	0
1	0	2	1	1	1	3	1	0	1	2
0	0	0	0	1	0	1	0	0	0	0
1	1	3	1	1	1	3	1	1	1	3
1	1	3	1	1	1	3	1	1	1	3
1	0	2	1	0	1	2	1	0	1	2
0	0	1	1	1	0	2	1	0	1	2
1	0	2	1	1	1	3	1	1	1	3
1	1	3	1	1	1	3	1	1	1	3
0	1	1	0	0	0	0	0	0	0	0

0	0	0	1	0	1	2	0	0	0	0
1	0	2	1	1	1	3	0	0	1	1
0	0	1	1	1	0	2	0	0	0	0
1	1	3	1	1	1	3	1	1	1	3
1	0	2	1	1	1	3	1	1	1	3
0	0	1	0	0	0	0	0	0	0	0
1	0	2	1	0	1	2	0	0	1	1
0	0	0	0	0	0	0	0	0	0	0
1	0	2	1	1	1	3	0	0	1	1
1	0	2	1	1	1	3	0	1	1	2
1	0	2	1	1	1	3	0	0	1	1
1	0	2	1	1	1	3	0	0	1	1
1	0	2	1	0	1	2	0	0	1	1
1	0	2	1	1	1	3	0	0	1	1
1	1	3	0	0	0	0	0	0	0	0
1	1	3	0	0	0	0	0	1	0	1
1	0	2	0	0	0	0	0	0	0	0
1	0	2	1	0	1	2	0	0	1	1
1	0	1	0	1	1	2	0	0	0	0
1	1	3	0	0	0	0	0	0	0	0
1	0	2	0	0	0	0	0	0	0	0
1	0	2	1	1	0	2	0	0	1	1
1	1	3	0	0	0	0	0	0	0	0
1	1	3	1	1	1	3	1	1	1	3
1	1	3	1	1	1	3	1	1	1	3
0	0	0	0	0	1	1	0	1	1	2
1	0	2	1	1	1	3	0	1	1	2
1	0	2	1	1	1	3	0	1	0	1
1	1	3	1	1	1	3	1	1	1	3
1	1	3	1	1	1	3	1	1	1	3
0	0	0	0	0	0	0	1	0	0	1
0	0	0	0	0	0	0	0	0	0	0
1	0	2	1	1	1	3	0	1	1	2
0	0	0	0	0	0	0	0	0	0	0
1	0	1	0	0	0	0	0	0	0	0
0	0	1	1	1	1	3	1	1	1	3
0	0	0	0	0	0	0	0	0	0	0
0	0	0	0	0	0	0	0	0	0	0
0	0	0	1	1	1	3	0	1	1	2
1	1	2	0	1	1	2	0	1	1	2
1	1	3	1	1	1	3	1	1	1	3
1	1	3	1	1	1	3	1	1	1	3
1	1	3	1	1	1	3	1	1	1	3
1	1	3	1	1	1	3	1	1	1	3
1	1	3	0	0	0	0	0	0	1	1
0	0	0	0	0	0	0	0	0	0	0
0	1	1	0	0	1	1	0	1	0	1
0	0	0	0	0	0	0	0	0	0	0
1	1	3	1	1	1	3	1	1	1	3
1	1	3	1	1	1	3	1	1	1	3
1	1	3	1	1	1	3	1	1	1	3
1	1	3	0	0	0	0	0	0	0	0
0	0	0	0	0	1	1	1	0	1	2
0	0	0	0	0	0	0	1	0	0	1
1	0	1	0	0	0	0	0	0	0	0
0	0	0	0	0	1	1	1	0	1	2
0	0	0	0	0	0	0	1	0	0	1
1	0	2	0	0	1	1	1	0	1	2
0	0	0	0	0	0	0	1	0	0	1

0	0	0	0	0	1	1	1	0	1	2
1	1	3	0	0	1	1	1	0	1	2
0	0	0	0	0	1	1	1	0	1	2
0	0	0	0	0	0	0	1	0	0	1
0	0	0	0	0	1	1	1	0	1	2
0	0	0	0	0	1	1	1	0	0	1
0	0	0	0	0	0	0	1	0	0	1
0	0	0	0	0	0	0	1	0	0	1
1	1	3	1	1	1	3	1	1	1	3
0	0	0	0	1	0	1	0	1	0	1
1	0	1	1	1	1	3	0	1	0	1
1	0	1	0	1	0	1	0	1	0	1
1	1	2	0	1	0	1	0	1	0	1
1	1	3	1	1	1	3	1	1	1	3
0	0	0	1	1	1	3	0	0	0	0
0	0	0	0	0	0	0	0	1	0	1
0	0	0	0	0	0	0	0	1	0	1
0	0	0	0	1	0	1	0	1	0	1
0	0	0	1	1	1	3	1	0	1	2
0	0	0	1	1	1	3	1	0	1	2
0	0	0	0	1	0	1	0	1	0	1
0	0	0	1	1	0	2	0	0	0	0
1	1	2	0	1	0	1	0	1	0	1
0	0	0	1	1	1	3	1	1	1	3
1	1	3	0	0	0	0	0	0	0	0
0	0	0	0	0	0	0	0	0	0	0
0	0	1	1	1	1	3	1	0	1	2
1	1	2	1	1	1	3	1	1	1	3
0	0	1	1	0	1	2	1	0	1	2
0	0	0	1	1	1	3	1	0	1	2
1	1	2	1	1	1	3	0	1	1	2
1	1	3	1	1	1	3	1	1	1	3
0	0	1	1	1	1	3	1	0	1	2
0	0	1	1	0	0	1	1	0	1	2
0	0	1	1	1	1	3	1	0	1	2
1	0	2	1	1	1	3	1	0	1	2
1	0	2	0	1	1	2	1	0	1	2
1	0	2	1	1	1	3	1	0	1	2
0	0	0	0	0	0	0	1	0	1	2
1	1	3	1	1	0	2	0	1	0	1
0	0	1	0	0	0	0	0	0	0	0
1	1	3	0	0	0	0	0	0	1	1
0	0	1	1	0	1	2	1	0	1	2
1	1	3	0	0	0	0	0	0	0	0
1	1	3	1	1	0	2	0	1	1	2
1	1	3	0	0	0	0	0	0	0	0
0	0	0	1	0	1	2	1	0	1	2
0	0	1	0	0	0	0	0	0	0	0
0	0	0	1	0	1	2	0	0	0	0
0	0	1	0	0	0	0	1	0	1	2
1	1	3	1	1	1	3	1	1	1	3
1	1	3	0	0	0	0	0	0	0	0
0	0	0	1	1	1	3	1	1	1	3
1	1	3	0	0	0	0	0	0	0	0
0	0	0	0	0	0	0	0	0	0	0
1	0	2	1	1	1	3	1	1	1	3
1	1	3	1	1	1	3	1	1	1	3
1	1	3	1	1	1	3	1	1	1	3
1	1	3	1	1	1	3	1	1	1	3

0	0	1	0	0	0	0	0	0	0	0
1	1	3	1	1	1	3	1	1	1	3
1	1	3	1	1	1	3	1	1	1	3
0	0	0	0	0	0	0	0	0	0	0
0	0	0	0	1	1	2	0	0	0	0
0	0	0	1	0	0	1	0	0	0	0
0	1	1	0	1	1	2	0	1	1	2
0	1	1	0	1	1	2	0	0	0	0
0	0	0	0	0	0	0	0	0	0	0
1	1	3	1	1	1	3	0	1	1	2
1	1	3	1	1	1	3	0	1	1	2
0	0	0	0	0	0	0	0	0	0	0
0	1	1	0	1	1	2	0	0	0	0
1	1	3	1	1	1	3	1	1	1	3
0	0	0	0	0	0	0	0	0	0	0
0	1	1	0	1	1	2	0	1	1	2
1	1	3	0	1	1	2	0	1	1	2
1	1	3	0	0	0	0	0	0	0	0
1	1	3	1	1	1	3	1	1	1	3
0	0	1	1	1	1	3	1	0	0	1
1	1	3	0	0	0	0	0	0	0	0
1	1	2	0	0	0	0	0	1	0	1
0	1	2	1	0	1	2	1	0	0	1
1	1	3	1	1	1	3	1	1	1	3
1	0	2	1	1	1	3	0	0	1	1
1	1	3	1	1	1	3	0	1	1	2
1	0	1	0	1	0	1	0	1	0	1
0	0	1	1	0	1	2	1	0	0	1
0	0	0	0	0	0	0	0	0	1	1
1	1	2	0	0	0	0	0	1	0	1
1	0	1	0	1	0	1	0	1	0	1
1	0	2	1	1	1	3	0	1	1	2
1	1	3	1	1	1	3	1	1	1	3
1	1	3	1	0	0	1	0	0	1	1
1	0	1	0	1	1	2	0	0	0	0
1	1	2	1	1	1	3	0	1	1	2
0	0	1	1	0	0	1	0	0	1	1
1	0	2	1	1	1	3	1	0	1	2
1	0	2	1	0	1	2	1	0	1	2
1	1	3	1	1	1	3	0	1	0	1
1	0	2	1	1	1	3	1	0	1	2
1	0	1	0	1	1	2	0	0	1	1
0	0	1	0	0	0	0	0	0	0	0
1	0	2	1	0	1	2	0	0	1	1
1	1	3	0	0	0	0	0	0	0	0
0	1	1	0	0	0	0	0	0	0	0
1	0	2	1	1	1	3	0	0	1	1
1	0	1	1	0	1	2	0	0	1	1
1	1	3	1	0	1	2	0	1	1	2
0	0	0	1	0	1	2	0	0	1	1
1	0	2	1	0	1	2	0	0	1	1
1	1	3	0	0	0	0	0	0	0	0
0	0	1	1	0	1	2	0	0	1	1
1	1	3	0	0	0	0	0	0	0	0
1	1	3	1	1	0	2	0	1	1	2
1	1	3	0	0	0	0	0	0	0	0
1	0	2	0	0	0	0	0	0	0	0
1	0	2	0	0	0	0	0	0	0	0
1	0	2	1	1	1	3	1	1	1	3

1	1	3	1	1	1	3	1	1	1	3
0	0	0	0	0	0	0	0	0	0	0
1	1	3	0	0	0	0	0	0	0	0
1	1	3	0	0	0	0	1	1	1	3
0	0	0	1	1	1	3	0	0	0	0
1	1	3	0	0	0	0	1	1	1	3
0	0	0	1	1	1	3	0	1	1	2
0	0	0	0	0	0	0	1	1	1	3
1	1	3	1	1	1	3	1	1	1	3
0	0	0	0	1	1	2	1	0	0	1
0	0	0	1	1	1	3	0	0	0	0
1	0	2	0	0	0	0	0	0	0	0
1	0	2	1	0	0	1	0	0	0	0
0	0	0	0	1	0	1	0	1	0	1
0	0	0	0	0	0	0	0	0	0	0
1	1	3	1	1	1	3	1	1	1	3
0	0	1	0	0	0	0	0	0	0	0
1	1	3	1	1	1	3	1	1	1	3
0	0	0	0	0	0	0	1	0	1	2
1	1	3	1	1	1	3	1	1	1	3
1	0	2	0	0	0	0	0	0	0	0
0	0	0	0	0	0	0	0	0	0	0
0	0	0	1	1	0	2	0	0	0	0
1	1	3	1	1	1	3	1	1	1	3
1	1	3	1	1	1	3	1	1	1	3
0	0	0	0	0	0	0	1	0	1	2
1	1	3	1	1	1	3	1	1	1	3
1	1	3	1	1	1	3	1	1	1	3
0	0	1	0	0	0	0	0	0	0	0
1	1	3	1	1	1	3	1	1	1	3
1	1	3	1	1	1	3	1	1	1	3
0	0	1	0	0	0	0	0	0	0	0
1	1	3	1	1	1	3	1	1	1	3
1	1	3	1	1	1	3	1	1	1	3
1	1	2	1	1	1	3	1	1	1	3
1	1	2	1	1	1	3	1	1	1	3
1	1	2	0	1	1	2	1	1	1	3
1	1	3	1	1	1	3	1	1	1	3
1	1	2	1	1	1	3	1	1	1	3
1	1	2	1	1	1	3	0	0	1	1
1	1	2	1	1	1	3	1	1	1	3
0	0	0	0	0	0	0	1	0	0	1
1	1	2	0	0	0	0	0	0	0	0
0	0	0	0	0	0	0	0	0	0	0
0	1	1	1	0	1	2	1	1	1	3
0	1	1	1	0	1	2	1	1	1	3
0	1	1	1	1	1	3	1	1	1	3
0	0	0	1	0	1	2	1	0	1	2
0	1	1	0	0	0	0	0	0	0	0
0	0	0	1	1	1	3	1	1	1	3
0	0	0	0	0	0	0	0	1	0	1
0	1	1	1	0	1	2	1	1	1	3
0	0	0	0	1	1	2	0	1	0	1
0	0	0	1	0	1	2	1	1	1	3
0	1	1	1	1	1	3	1	1	1	3
0	1	1	1	1	1	3	1	1	1	3
0	0	0	0	0	0	0	1	0	0	1
0	1	1	1	0	1	2	1	1	1	3
0	1	1	1	0	1	2	1	0	1	2

0	0	0	0	1	1	2	1	0	1	2
0	0	0	1	0	1	2	1	0	1	2
0	0	0	0	0	0	0	1	0	1	2
0	0	0	1	0	1	2	1	0	1	2
0	0	0	1	0	0	1	1	0	1	2
0	0	0	1	0	1	2	1	0	1	2
0	1	1	1	1	1	3	1	1	1	3
1	1	3	1	1	1	3	1	1	1	3
0	1	1	0	0	1	1	0	1	0	1
0	0	0	1	0	1	2	1	0	1	2
0	0	0	1	0	1	2	1	0	1	2
0	0	0	0	0	0	0	1	0	0	1
0	0	0	0	0	0	0	1	0	1	2
0	0	0	0	0	0	0	1	0	1	2
0	1	1	1	1	1	3	1	1	1	3
0	1	1	1	0	0	1	0	0	0	0
0	1	1	1	0	1	2	1	1	1	3
0	1	1	1	0	1	2	1	1	1	3
0	0	0	0	0	0	0	0	0	0	0
0	1	1	1	1	1	3	1	1	1	3
0	0	0	0	0	0	0	0	0	0	0
0	1	1	0	0	1	1	1	0	0	1
0	1	1	0	0	1	1	0	1	0	1
0	0	0	0	0	0	0	0	0	0	0
0	1	1	0	0	0	0	0	0	0	0
0	1	1	1	0	1	2	1	1	1	3
0	0	0	0	0	0	0	0	0	0	0
0	1	1	0	0	0	0	0	0	0	0
0	0	1	1	0	0	1	0	0	0	0
1	0	1	0	1	0	1	0	0	0	0
1	1	3	1	1	1	3	1	1	1	3
1	1	3	1	1	1	3	1	1	1	3
1	0	2	1	1	1	3	1	1	1	3
1	1	3	0	0	0	0	0	0	0	0
1	1	3	1	1	1	3	1	1	1	3
1	1	3	1	1	1	3	1	1	1	3
1	1	2	1	1	1	3	0	1	1	2
0	0	0	0	0	0	0	0	0	0	0
1	1	3	0	0	0	0	0	0	0	0
1	1	3	0	0	0	0	0	1	1	2
1	1	2	1	1	1	3	1	0	1	2
0	1	1	0	0	0	0	1	1	1	3
1	0	2	0	1	1	2	0	0	0	0
0	1	1	1	0	0	1	1	1	1	3
0	0	1	0	1	1	2	0	0	0	0
0	1	1	1	0	0	1	1	1	1	3
1	0	2	0	1	1	2	0	0	0	0
0	0	0	1	0	0	1	1	0	0	1
1	0	2	0	1	1	2	0	0	0	0
0	1	1	1	0	0	1	1	1	1	3
1	0	2	0	1	0	1	0	0	0	0
1	0	2	0	0	0	0	0	0	0	0
0	0	0	0	0	0	0	0	0	1	1
0	0	0	0	0	0	0	0	0	0	0
0	0	0	0	0	0	0	0	0	0	0
0	0	0	1	0	1	2	0	1	1	2
0	0	0	1	0	1	2	0	1	1	2
1	0	1	1	1	1	3	0	1	1	2
0	0	0	0	0	1	1	0	0	1	1

0	0	0	0	0	0	0	0	1	0	1
0	1	1	0	1	1	2	0	1	1	2
0	0	0	0	1	1	2	0	0	0	0
0	0	0	1	1	1	3	0	1	1	2
1	1	2	0	0	0	0	0	0	0	0
1	1	3	1	1	1	3	1	1	1	3
0	0	0	0	0	0	0	0	0	0	0
1	1	3	1	1	1	3	1	1	1	3
1	1	3	1	0	1	2	0	1	1	2
1	1	2	0	0	0	0	0	0	0	0
1	1	2	0	0	0	0	0	0	0	0
1	1	3	1	1	1	3	0	1	1	2
1	1	2	0	1	1	2	0	1	1	2
0	0	0	0	0	1	1	0	1	1	2
0	0	0	0	0	1	1	0	1	0	1
0	0	0	1	0	1	2	0	1	1	2
1	1	3	1	1	1	3	1	1	1	3
1	1	2	0	0	0	0	0	0	1	1
1	1	2	0	0	0	0	0	0	0	0

Strain 5271

										Msp I
(R = 20°C)			(C = 20°C)				(S = 4°C)			
R2	R3	sum	C1	C2	C3	sum	S1	S2	S3	sum
1	1	3	1	1	1	3	1	1	1	3
0	1	1	0	0	0	0	0	0	0	0
0	0	0	1	1	1	3	1	0	1	2
0	1	1	1	0	1	2	1	0	1	2
0	0	0	1	1	1	3	0	1	1	2
0	1	2	1	1	1	3	1	1	1	3
1	1	3	1	1	1	3	1	1	1	3
0	0	0	0	1	0	1	0	1	0	1
1	1	3	1	1	1	3	1	1	1	3
0	0	0	0	1	0	1	0	1	0	1
1	1	3	1	0	1	2	1	0	1	2
0	1	1	0	0	0	0	0	0	1	1
0	0	0	1	0	1	2	1	0	1	2
0	0	0	0	1	0	1	0	0	1	1
1	1	3	1	1	1	3	1	1	1	3
1	1	3	1	1	1	3	1	1	1	3
0	0	0	0	1	0	1	0	1	0	1
0	1	1	1	1	1	3	1	1	1	3
1	1	3	1	1	1	3	1	1	1	3
0	0	0	1	1	1	3	1	1	1	3
0	0	0	0	0	0	0	0	1	0	1
0	0	0	0	1	0	1	0	1	0	1
1	1	3	1	1	1	3	1	1	1	3
1	1	3	1	1	1	3	1	1	1	3
0	0	0	0	0	0	0	0	0	0	0
1	1	3	1	1	1	3	1	0	1	2
0	0	0	0	0	0	0	0	1	0	1
1	1	3	1	0	1	2	1	1	1	3
0	1	1	0	1	0	1	0	1	0	1
0	0	0	0	1	0	1	0	1	0	1
0	0	0	0	1	0	1	0	1	0	1
1	1	3	1	0	1	2	1	0	1	2
0	1	1	0	0	0	0	0	0	1	1

0	0	0	0	0	0	0	0	0	0	0
0	0	0	0	1	0	1	0	1	0	1
1	1	3	0	0	0	0	0	0	0	0
1	1	3	1	1	1	3	0	0	1	1
0	1	2	0	1	1	2	0	0	0	0
0	0	0	0	1	0	1	0	1	0	1
0	0	0	0	1	0	1	0	1	0	1
1	1	3	1	1	1	3	1	1	1	3
0	0	0	0	1	0	1	0	1	0	1
1	1	3	1	1	1	3	1	1	1	3
1	1	3	1	1	1	3	1	1	1	3
0	0	0	0	0	0	0	0	0	0	0
1	1	3	0	0	0	0	0	0	0	0
0	0	0	1	1	1	3	1	0	1	2
0	0	0	1	0	1	2	1	0	0	1
0	0	0	0	0	0	0	0	0	0	0
0	0	0	0	1	0	1	0	1	0	1
0	0	0	0	0	0	0	0	0	0	0
1	1	3	1	1	1	3	1	1	0	2
1	0	2	1	1	1	3	1	0	0	1
0	0	0	0	0	0	0	0	1	0	1
0	0	0	0	0	0	0	0	1	0	1
0	0	0	0	1	0	1	0	1	0	1
1	1	3	1	0	1	2	1	0	0	1
0	0	0	1	1	1	3	1	0	0	1
1	1	3	0	0	0	0	1	0	1	2
0	0	0	0	0	0	0	0	0	0	0
0	0	0	0	1	0	1	0	1	0	1
1	1	3	1	1	1	3	1	1	1	3
1	0	1	0	0	0	0	0	0	0	0
1	1	3	1	1	1	3	1	0	1	2
0	0	0	0	1	0	1	0	1	0	1
1	1	3	0	0	0	0	0	0	0	0
0	0	0	0	0	0	0	0	0	0	0
1	1	3	1	0	0	1	1	0	1	2
1	1	3	1	1	1	3	1	1	1	3
1	1	3	1	0	0	1	1	1	1	3
1	1	2	1	0	0	1	0	0	0	0
1	1	3	1	0	0	1	1	1	1	3
1	1	3	1	0	0	1	1	0	1	2
0	0	1	0	0	0	0	0	1	0	1
0	0	0	1	0	0	1	1	0	1	2
1	1	3	0	0	0	0	0	1	0	1
0	0	0	1	0	0	1	1	1	1	3
1	1	3	0	0	0	0	1	0	1	2
0	0	0	0	0	0	0	0	0	1	1
0	0	0	0	1	0	1	0	1	0	1
0	0	0	0	1	0	1	1	1	1	3
0	0	0	0	0	0	0	0	1	0	1
0	0	0	1	0	0	1	1	0	1	2
1	1	3	0	0	0	0	0	0	0	0
1	1	3	0	0	0	0	1	0	1	2
0	0	0	0	0	0	0	0	0	0	0
0	0	0	0	1	0	1	0	1	0	1
0	0	0	0	1	0	1	0	0	0	0
1	1	3	0	0	0	0	0	0	0	0
0	0	0	1	0	0	1	0	1	0	1
0	0	0	1	1	1	3	1	1	1	3
0	0	0	0	0	0	0	0	1	0	1

0	0	0	0	1	0	1	0	1	0	1
0	0	0	0	0	0	0	0	1	0	1
0	0	0	0	0	0	0	0	0	0	0
1	1	3	1	0	0	1	1	0	1	2
1	1	3	0	0	0	0	0	0	1	1
1	1	3	0	0	0	0	0	0	0	0
0	0	0	0	0	0	0	0	0	0	0
0	0	0	0	0	0	0	0	0	0	0
0	0	0	0	0	0	0	0	0	0	0
0	0	0	0	0	0	0	0	0	0	0
1	1	3	0	1	0	1	0	0	1	1
1	1	3	1	0	0	1	1	0	1	2
0	0	0	0	0	0	0	0	1	0	1
1	1	3	1	1	1	3	1	1	1	3
1	1	3	1	1	1	3	1	1	1	3
0	0	0	0	1	0	1	0	1	0	1
0	0	0	0	1	0	1	0	1	0	1
0	0	0	0	1	0	1	0	1	0	1
1	1	3	1	1	1	3	1	1	1	3
0	1	2	1	1	1	3	1	0	1	2
0	0	0	0	1	0	1	0	1	0	1
1	1	3	1	1	1	3	1	1	1	3
1	1	3	0	1	0	1	0	1	1	2
0	0	0	0	1	0	1	0	1	0	1
0	0	0	1	1	1	3	1	0	1	2
0	0	0	1	0	1	2	0	0	1	1
0	0	0	0	1	0	1	0	1	0	1
1	1	3	1	0	1	2	0	0	1	1
1	1	3	1	0	1	2	0	1	1	2
0	1	2	0	0	0	0	0	0	0	0
0	0	0	0	0	0	0	0	0	0	0
0	0	0	0	0	0	0	0	1	0	1
0	0	0	0	0	0	0	0	1	0	1
0	0	0	0	1	0	1	0	1	0	1
0	0	0	0	1	0	1	0	1	0	1
0	0	0	0	0	0	0	0	0	0	0
0	0	1	1	0	1	2	1	0	1	2
1	1	3	1	0	1	2	0	0	1	1
0	0	0	0	0	0	0	0	1	0	1
0	0	0	1	1	0	2	0	1	0	1
1	1	3	1	1	1	3	1	1	1	3
1	1	3	1	0	1	2	0	0	1	1
1	1	3	0	0	0	0	0	0	1	1
1	1	3	0	0	0	0	0	0	1	1
0	1	1	0	0	1	1	0	0	1	1
0	0	0	0	0	0	0	0	1	0	1
1	0	2	1	0	0	1	0	1	0	1
1	1	3	1	0	1	2	0	0	1	1
1	1	3	1	1	1	3	1	1	1	3
0	0	0	0	0	0	0	0	1	0	1
1	0	2	1	0	0	1	1	0	1	2
1	1	3	1	0	1	2	0	0	1	1
0	0	0	0	0	0	0	0	1	0	1
1	1	3	0	0	0	0	0	0	1	1
0	0	0	0	0	0	0	0	1	0	1
0	0	0	0	0	0	0	0	1	0	1
1	1	3	0	0	0	0	0	0	0	0
1	1	3	0	0	0	0	0	0	1	1
1	1	3	0	0	0	0	0	0	0	0

1	1	3	1	0	1	2	0	0	1	1
0	0	1	0	0	1	1	0	0	0	0
0	0	0	0	0	0	0	0	1	0	1
0	0	0	1	1	1	3	1	1	1	3
1	1	3	0	0	0	0	0	0	0	0
1	1	3	1	0	1	2	0	0	1	1
0	0	0	0	0	0	0	0	1	0	1
0	0	0	0	0	0	0	0	1	0	1
1	0	2	1	0	1	2	0	0	1	1
0	0	0	0	0	0	0	0	1	0	1
0	0	0	0	0	0	0	0	0	0	0
1	1	3	0	0	0	0	0	0	1	1
0	0	0	0	0	0	0	0	1	0	1
0	0	0	0	0	0	0	0	1	0	1
0	0	0	0	0	0	0	0	0	0	0
1	1	3	0	0	0	0	0	0	0	0
1	1	3	0	0	0	0	0	0	0	0
0	0	0	0	0	0	0	0	0	0	0
1	1	3	1	0	1	2	0	0	1	1
1	1	3	0	0	1	1	0	0	1	1
1	1	3	0	0	0	0	0	0	0	0
1	1	3	0	0	0	0	0	0	1	1
1	1	3	1	1	1	3	1	1	1	3
1	1	3	1	0	1	2	1	0	1	2
1	1	3	1	1	1	3	1	1	1	3
0	0	0	1	0	1	2	1	0	1	2
0	0	0	0	1	0	1	1	1	1	3
0	1	1	1	0	1	2	1	0	1	2
0	0	0	0	1	0	1	0	1	0	1
0	0	0	1	0	0	1	1	0	1	2
0	0	0	0	0	0	0	0	1	0	1
0	0	0	0	1	0	1	0	1	0	1
1	1	3	1	1	1	3	1	1	1	3
0	0	0	0	0	0	0	0	0	0	0
0	0	0	0	1	0	1	0	1	1	2
0	0	0	0	0	0	0	0	0	0	0
0	1	1	1	1	1	3	1	1	1	3
1	1	3	1	1	1	3	1	1	1	3
1	1	3	1	1	1	3	1	1	1	3
1	1	3	1	0	0	1	0	0	0	0
0	0	0	0	1	0	1	0	1	0	1
1	1	3	1	0	1	2	1	0	1	2
0	0	0	0	1	0	1	0	1	0	1
1	0	2	1	0	0	1	1	1	1	3
1	1	3	1	1	1	3	1	1	1	3
0	0	0	0	1	0	1	0	1	0	1
1	1	3	1	0	1	2	1	0	1	2
1	1	3	1	1	1	3	1	1	1	3
0	0	0	0	1	0	1	0	1	0	1
1	1	3	1	0	1	2	1	0	1	2
0	0	0	0	0	0	0	0	0	0	0
1	1	3	0	0	0	0	0	0	0	0
0	0	0	0	1	0	1	0	1	0	1
0	0	0	0	1	0	1	0	1	0	1
0	0	0	0	1	0	1	0	1	0	1
0	0	0	0	0	0	0	0	0	0	0
0	0	0	0	0	0	0	0	0	0	0
1	1	3	0	0	0	0	0	0	0	0
1	1	2	1	0	1	2	1	0	1	2

1	0	1	1	0	0	1	0	0	1	1
0	0	0	0	0	0	0	0	0	0	0
1	1	3	1	0	1	2	1	0	1	2
0	0	0	0	0	0	0	0	0	0	0
0	0	0	0	0	0	0	0	0	0	0
0	0	0	0	0	0	0	0	0	0	0
0	0	0	0	0	0	0	0	0	0	0
0	0	0	0	0	0	0	0	0	0	0
0	0	0	1	0	0	1	1	0	1	2
0	0	0	0	0	0	0	0	0	0	0
0	0	0	0	0	0	0	0	0	0	0
1	1	3	1	1	1	3	1	1	1	3
1	1	3	1	1	1	3	1	1	1	3
0	0	0	1	1	1	3	0	1	1	2
0	1	1	1	1	1	3	1	0	1	2
0	0	0	0	0	0	0	0	1	0	1
0	1	2	1	1	1	3	1	0	1	2
1	1	3	1	1	1	3	1	1	1	3
0	0	0	0	1	0	1	0	1	0	1
1	1	3	1	1	1	3	1	1	1	3
0	0	0	0	1	0	1	0	1	0	1
1	1	3	1	1	1	3	1	1	1	3
1	1	3	1	1	1	3	1	1	1	3
0	0	0	1	1	1	3	1	0	1	2
1	1	3	1	0	1	2	0	1	1	2
0	0	1	0	0	0	0	0	0	0	0
1	1	3	0	1	0	1	0	0	0	0
0	0	0	0	0	0	0	0	1	0	1
0	0	0	1	1	1	3	1	0	1	2
0	0	0	0	0	0	0	0	0	0	0
0	0	0	0	0	0	0	0	0	0	0
1	1	3	0	0	1	1	0	1	1	2
1	1	3	0	0	0	0	0	1	0	1
0	0	0	0	0	0	0	0	0	0	0
0	0	0	0	0	0	0	0	0	0	0
0	0	0	0	1	0	1	0	1	0	1
0	0	0	0	1	0	1	0	1	0	1
0	0	0	0	0	0	0	0	0	0	0
0	0	0	0	1	0	1	0	1	0	1
1	1	3	1	1	1	3	1	1	1	3
0	0	0	0	0	0	0	0	0	0	0
0	0	0	0	1	0	1	0	1	0	1
0	0	0	0	1	0	1	0	1	0	1
0	0	0	1	0	0	1	0	0	0	0
0	0	0	0	0	0	0	0	1	0	1
1	1	3	0	0	0	0	0	0	0	0
0	0	0	0	0	0	0	0	0	0	0
0	0	0	0	0	0	0	0	0	0	0
0	0	0	0	0	0	0	0	0	0	0
0	0	0	0	0	0	0	0	0	0	0
0	0	0	0	0	0	0	0	0	0	0
1	1	3	1	1	1	3	1	1	1	3
1	1	3	1	1	1	3	1	1	1	3
1	1	3	1	1	1	3	1	1	1	3
0	0	0	0	1	0	1	0	1	0	1
1	1	3	0	0	0	0	0	0	0	0
0	0	0	0	0	0	0	0	0	0	0
0	0	0	0	0	0	0	0	0	0	0
1	1	3	1	0	0	1	1	0	0	1

0	1	1	1	0	1	2	1	0	0	1
0	1	2	1	0	0	1	1	0	1	2
0	0	0	1	0	1	2	1	0	1	2
0	0	0	0	1	1	2	0	1	0	1
1	1	3	1	0	1	2	1	0	1	2
0	0	0	1	0	1	2	1	0	1	2
0	0	0	0	1	0	1	0	0	1	1
1	1	3	1	1	1	3	1	1	1	3
0	0	0	0	1	0	1	0	1	0	1
0	0	1	0	0	0	0	0	0	0	0
1	1	3	1	1	1	3	1	0	1	2
0	0	0	0	1	0	1	0	1	1	2
1	1	3	1	1	1	3	1	1	1	3
0	1	1	1	0	1	2	1	0	1	2
0	0	0	0	1	0	1	0	0	0	0
0	0	0	1	0	1	2	1	0	1	2
1	1	3	1	0	1	2	1	0	1	2
1	1	3	0	0	0	0	0	0	0	0
1	1	3	1	0	1	2	1	0	1	2
0	0	0	0	0	0	0	0	1	0	1
0	0	0	0	0	0	0	0	1	0	1
1	1	3	1	1	1	3	1	1	1	3
1	1	3	1	0	1	2	1	0	1	2
1	0	2	1	0	0	1	1	0	0	1
1	1	3	0	0	0	0	0	0	0	0
0	0	0	0	1	1	2	0	1	0	1
0	0	0	0	1	1	2	0	1	0	1
1	1	3	1	0	1	2	1	0	1	2
0	0	0	0	0	0	0	0	0	0	0
1	1	3	1	0	1	2	1	0	1	2
0	0	0	0	1	0	1	0	1	0	1
0	0	0	0	1	0	1	0	1	0	1
1	1	3	0	0	0	0	0	0	0	0
0	0	0	0	1	0	1	0	1	0	1
1	1	3	1	1	1	3	1	0	1	2
1	1	3	1	0	1	2	1	0	1	2
1	1	3	0	0	0	0	0	0	0	0
1	1	3	1	1	1	3	1	0	1	2
1	1	3	0	0	0	0	0	0	0	0
0	0	0	1	0	0	1	1	0	1	2
0	0	0	0	0	0	0	0	0	0	0
1	1	3	1	1	1	3	1	1	1	3
0	0	0	0	0	0	0	0	0	0	0
0	1	1	0	0	0	0	0	0	0	0
1	1	3	0	1	0	1	0	1	1	2
0	0	0	0	0	0	0	0	0	0	0
1	1	3	1	1	1	3	1	1	1	3
0	0	0	0	0	0	0	0	0	0	0
0	0	0	0	1	0	1	0	1	0	1
0	0	0	0	1	0	1	0	1	0	1
0	0	0	0	0	0	0	1	1	0	2
0	0	0	0	0	0	0	0	0	0	0
0	1	2	1	1	1	3	1	0	1	2
0	0	0	0	1	0	1	0	0	0	0
1	1	3	0	0	0	0	0	1	0	1
0	0	0	0	0	0	0	0	1	0	1
0	0	0	0	1	0	1	0	1	0	1
0	0	0	0	1	0	1	0	1	0	1
0	0	0	0	0	0	0	1	0	0	1

0	0	0	0	0	0	0	0	1	0	1
0	1	1	0	1	0	1	0	1	0	1
0	0	0	0	0	0	0	0	1	0	1
0	0	0	0	0	0	0	0	1	0	1
0	0	0	0	0	0	0	0	1	0	1
0	0	0	0	1	0	1	0	1	0	1
0	0	0	0	0	0	0	0	1	0	1
0	0	0	0	0	0	0	0	1	0	1
1	1	3	1	1	1	3	1	0	1	2
0	0	0	0	0	0	0	0	1	0	1
1	1	3	0	0	0	0	0	0	0	0
0	0	0	0	0	0	0	0	0	0	0
1	1	3	1	1	1	3	1	0	1	2
0	0	0	0	0	0	0	0	1	0	1
0	0	0	0	0	0	0	0	1	0	1
1	1	3	0	0	0	0	0	0	0	0
1	1	3	1	0	1	2	1	0	1	2
0	0	0	0	0	0	0	0	1	0	1
1	1	3	1	1	1	3	1	1	1	3
0	0	0	0	0	0	0	0	0	0	0
0	0	0	0	0	0	0	0	0	0	0
1	1	3	1	1	1	3	1	1	1	3
0	0	0	1	0	1	2	0	0	1	1
0	0	0	1	0	1	2	1	0	1	2
0	0	0	0	0	0	0	0	1	0	1
1	1	3	1	1	1	3	1	0	1	2
1	1	3	1	0	1	2	1	0	1	2
0	0	0	0	0	0	0	0	0	0	0
1	1	3	1	1	1	3	1	1	1	3
1	1	3	0	0	0	0	0	0	0	0
1	1	3	1	1	1	3	1	1	1	3
1	1	3	1	0	1	2	0	0	1	1
1	1	3	0	0	0	0	0	0	0	0
0	0	0	1	1	0	2	0	1	0	1
1	1	3	1	1	1	3	1	1	1	3
1	1	3	1	1	1	3	1	1	1	3
1	1	3	1	1	1	3	1	1	1	3
1	1	3	1	1	1	3	1	1	1	3
0	0	0	0	0	0	0	0	0	0	0
0	0	0	1	0	1	2	1	0	1	2
0	0	0	0	0	0	0	0	1	0	1
0	0	0	0	0	0	0	0	0	0	0
0	0	0	1	0	1	2	1	1	1	3
0	0	0	1	1	0	2	0	1	1	2
0	0	0	0	0	0	0	0	1	0	1
0	0	0	0	1	0	1	0	1	0	1
0	0	0	0	0	0	0	0	0	0	0
0	0	0	0	0	0	0	0	0	0	0
1	1	3	0	0	0	0	0	0	0	0
1	1	3	0	0	0	0	0	1	0	1
0	0	0	0	0	0	0	0	1	1	2
0	0	0	0	0	0	0	0	1	0	1
0	0	0	0	1	0	1	0	1	0	1
0	0	0	0	0	0	0	0	0	0	0
1	0	2	1	1	1	3	1	1	1	3
0	0	0	0	0	0	0	0	0	0	0
1	1	3	1	1	1	3	1	1	1	3
1	1	3	1	1	1	3	1	1	1	3
1	1	3	0	0	1	1	1	0	0	1

0	0	0	0	0	0	0	0	1	0	1
1	1	3	1	1	1	3	1	1	1	3
0	1	1	1	0	1	2	1	0	1	2
1	1	3	1	1	1	3	1	1	1	3
0	0	0	0	1	0	1	0	1	0	1
1	1	3	1	1	1	3	1	1	1	3
1	1	3	1	1	1	3	1	1	1	3
1	1	3	1	1	1	3	1	1	1	3
0	0	0	0	1	0	1	0	1	0	1
0	0	0	0	0	0	0	0	1	0	1
0	0	0	0	0	0	0	0	0	0	0
0	0	0	0	0	0	0	0	1	0	1
0	0	0	0	0	0	0	0	1	0	1
1	1	3	0	0	0	0	0	0	0	0
0	0	0	0	1	0	1	0	1	0	1
1	1	3	1	0	1	2	1	0	1	2
1	1	3	0	0	0	0	0	0	0	0
1	1	3	0	0	0	0	0	0	0	0
1	1	3	1	1	1	3	1	1	1	3
0	0	0	0	0	0	0	0	0	0	0
1	1	3	0	0	0	0	0	0	0	0
1	1	3	1	1	1	3	1	1	1	3
0	0	0	0	1	0	1	0	1	0	1
1	1	3	1	1	1	3	1	1	1	3
1	1	3	0	0	0	0	0	0	0	0
0	0	0	0	0	0	0	0	0	0	0
1	1	3	1	1	1	3	1	1	1	3
0	0	0	0	0	0	0	0	1	0	1
0	0	0	0	0	0	0	0	1	0	1
1	1	3	1	0	1	2	1	1	1	3
1	1	3	1	0	1	2	1	0	1	2
1	0	2	1	0	1	2	1	0	1	2
0	0	1	1	0	1	2	1	0	1	2
1	0	2	1	0	1	2	1	0	1	2
0	0	0	0	0	0	0	1	0	0	1
1	1	3	1	0	1	2	1	0	1	2
1	0	1	1	0	1	2	0	0	1	1
0	0	0	1	0	0	1	1	0	1	2
0	0	0	0	0	0	0	0	1	0	1
0	0	0	0	0	0	0	0	1	0	1
1	0	2	1	0	0	1	0	0	1	1
1	1	3	1	0	1	2	1	0	1	2
1	0	2	1	0	1	2	1	0	1	2
0	0	0	1	0	0	1	1	1	1	3
0	0	0	0	0	0	0	0	1	0	1
1	1	3	1	0	1	2	1	1	1	3
1	1	3	1	0	1	2	1	1	1	3
0	0	0	0	0	0	0	1	0	0	1
0	0	0	1	0	1	2	0	1	1	2
0	0	0	0	0	0	0	0	0	0	0
1	0	1	1	0	0	1	0	0	0	0
1	1	3	1	0	1	2	0	0	1	1
0	0	0	0	0	0	0	0	0	0	0
0	0	0	0	0	0	0	0	0	1	1
1	0	2	1	0	1	2	0	0	1	1
1	0	2	1	0	1	2	0	0	1	1
0	0	0	0	0	0	0	0	1	0	1
0	0	0	0	0	0	0	0	1	0	1
1	0	2	1	0	1	2	1	0	1	2

0	0	0	0	0	0	0	0	1	0	1
1	0	2	1	0	1	2	1	0	1	2
1	0	2	1	0	1	2	1	0	1	2
0	0	0	0	0	0	0	1	0	0	1
0	0	1	0	0	0	0	0	0	0	0
0	0	1	0	0	0	0	0	0	0	0
0	0	1	1	0	0	1	0	0	0	0
1	0	2	1	0	1	2	1	0	0	1
1	0	2	1	0	1	2	1	0	1	2
0	0	0	0	0	0	0	0	0	0	0
1	1	3	1	0	1	2	1	1	1	3
1	0	1	0	0	0	0	0	0	0	0
1	0	2	0	0	0	0	0	0	0	0
0	0	0	0	0	0	0	0	1	0	1
0	0	0	0	0	0	0	0	1	0	1
0	0	0	0	0	0	0	0	0	0	0
1	0	2	1	0	1	2	1	0	1	2
1	0	2	1	0	1	2	1	0	1	2
0	1	2	1	0	1	2	1	0	1	2
0	0	0	0	0	0	0	0	1	0	1
1	0	2	1	0	1	2	1	0	1	2
1	1	3	1	0	1	2	1	0	1	2
0	0	0	0	0	0	0	0	1	0	1
1	0	2	1	0	1	2	0	0	0	0
1	0	1	0	0	0	0	1	1	0	2
1	1	2	0	0	1	1	0	0	1	1
1	0	2	1	0	1	2	1	0	1	2
1	0	2	1	0	1	2	1	0	1	2
0	0	0	0	0	0	0	0	0	0	0
0	0	0	0	0	0	0	0	0	0	0
0	0	0	0	0	0	0	0	1	0	1
1	1	3	0	0	0	0	0	0	0	0
0	0	0	0	0	0	0	0	0	0	0
0	0	0	0	0	0	0	0	0	0	0
1	0	2	0	0	0	0	0	0	0	0
1	0	2	1	0	1	2	1	0	1	2
1	0	2	0	0	0	0	0	0	0	0
1	1	2	0	0	1	1	1	0	1	2
0	0	0	1	0	1	2	1	0	1	2
0	0	0	0	0	0	0	0	0	0	0
0	0	0	1	0	1	2	1	0	1	2
0	0	0	1	0	1	2	1	0	1	2
1	1	3	1	1	1	3	1	1	1	3
1	1	3	1	1	1	3	1	1	1	3
1	1	3	1	1	1	3	1	1	1	3
0	1	1	1	0	1	2	1	0	1	2
0	0	0	0	1	0	1	0	1	0	1
0	0	0	0	0	0	0	0	0	0	0
0	0	0	1	0	0	1	1	0	1	2
0	0	0	0	1	0	1	0	1	0	1
0	0	0	0	1	0	1	0	1	0	1
0	0	0	0	1	0	1	0	1	0	1
0	0	0	0	0	0	0	0	0	0	0
0	0	0	0	0	0	0	0	1	0	1
0	0	0	0	0	0	0	0	1	0	1
1	1	3	0	0	0	0	0	0	0	0
0	0	0	0	1	0	1	0	1	0	1
1	1	3	1	1	1	3	1	0	1	2
0	0	0	0	1	0	1	0	1	0	1

0	0	0	0	0	0	0	0	0	0	0
0	0	0	0	1	0	1	0	1	0	1
0	0	0	0	1	0	1	0	1	0	1
0	0	0	1	0	0	1	0	0	0	0
0	0	0	0	0	0	0	0	0	0	0
0	0	0	0	0	0	0	0	0	0	0
0	0	0	1	0	0	1	1	1	1	3
0	0	0	1	0	0	1	1	0	1	2
0	0	0	1	0	0	1	1	0	1	2
0	1	1	1	0	1	2	1	1	1	3
0	0	0	1	0	0	1	1	0	1	2
0	0	0	0	0	0	0	0	1	0	1
0	0	0	1	0	1	2	1	1	1	3
0	0	0	1	0	1	2	1	0	1	2
0	0	0	0	0	0	0	0	1	0	1
0	0	0	1	0	1	2	1	0	1	2
0	0	0	1	0	0	1	0	1	0	1
0	0	0	1	0	1	2	1	0	1	2
1	1	3	0	0	0	0	0	0	0	0
0	0	0	0	0	0	0	0	1	0	1
0	0	0	0	0	0	0	0	1	0	1
0	0	0	0	0	0	0	0	1	0	1
1	1	2	1	0	1	2	1	1	1	3
0	0	0	0	0	0	0	0	0	0	0
1	1	3	0	1	1	2	1	0	1	2
0	0	0	1	1	1	3	1	1	1	3
1	1	3	1	1	1	3	1	1	1	3
1	1	3	1	0	0	1	1	1	1	3
0	0	0	0	0	0	0	0	1	0	1
0	0	0	0	0	0	0	0	0	0	0
1	1	3	1	0	1	2	1	0	1	2
0	0	0	0	0	0	0	0	0	0	0
0	0	0	0	0	0	0	0	1	0	1
0	0	0	1	0	0	1	1	0	1	2
0	0	0	0	0	0	0	0	1	0	1
0	0	0	0	0	0	0	0	1	0	1
0	0	0	0	0	0	0	0	1	0	1
0	0	0	0	1	0	1	0	1	0	1
0	0	0	1	0	0	1	1	0	1	2
0	0	0	0	0	0	0	1	0	1	2
0	0	0	0	0	0	0	0	1	0	1
1	1	3	1	1	1	3	1	1	1	3
0	0	0	1	0	0	1	1	0	1	2
1	1	3	0	0	0	0	0	0	0	0
0	0	0	1	0	0	1	0	0	0	0

(R = 25°C)

R1	R2	R3	sum
1	1	1	3
0	0	0	0
1	1	1	3
0	0	0	0
1	1	1	3
1	1	1	3
1	1	1	3
1	1	1	3
1	1	1	3
1	1	1	3
1	1	1	3
1	1	1	3
0	0	0	0
1	1	1	3
1	1	1	3
1	1	1	3
1	1	1	3
0	0	1	1
1	1	1	3
0	0	0	0
1	1	1	3
1	1	1	3
0	0	0	0
0	0	0	0
0	0	0	0
1	1	1	3
0	0	0	0
1	1	1	3
1	1	1	3
0	0	0	0
0	0	0	0
1	1	1	3
1	1	1	3
1	1	1	3
0	0	0	0
0	0	0	0
0	0	0	0
0	1	1	2
1	0	1	2
1	1	1	3
1	1	1	3
1	1	1	3
0	0	0	0
0	0	0	0
1	1	1	3
0	0	0	0
0	0	0	0
1	1	1	3
0	0	0	0
0	0	0	0

1	1	1	3
1	1	1	3
0	0	1	1
1	1	1	3
1	1	1	3
1	1	1	3
0	0	0	0
1	1	1	3
1	1	1	3
1	1	1	3
1	1	1	3
1	1	1	3
0	0	0	0
0	0	0	0
1	1	1	3
1	1	1	3
1	1	1	3
0	0	0	0
1	1	1	3
1	1	1	3
1	1	1	3
1	1	1	3
0	0	0	0
1	1	1	3
1	1	1	3
1	1	1	3
1	1	1	3
0	0	0	0
1	1	1	3
0	0	0	0
1	1	1	3
0	0	0	0
0	0	1	1
0	0	0	0
1	1	1	3
1	1	1	3
0	0	0	0
1	1	1	3
1	1	1	3
1	1	0	2
1	1	1	3
1	1	0	2
1	1	1	3
1	1	0	2
0	0	1	1
1	1	1	3
1	1	0	2
1	1	0	2
0	0	0	0
0	0	0	0
1	0	1	2
0	0	0	0
1	1	1	3
1	1	1	3
0	0	1	1
0	0	0	0
1	1	1	3
1	1	1	3
1	1	0	2

0	0	1	1
1	0	1	2
0	0	1	1
1	1	1	3
1	1	1	3
0	0	1	1
1	0	1	2
0	0	0	0
1	0	1	2
1	1	1	3
0	0	1	1
1	1	1	3
1	0	1	2
1	0	1	2
0	0	0	0
0	0	0	0
0	0	0	0
0	0	1	1
0	0	1	1
0	0	0	0
0	0	0	0
0	0	1	1
0	0	0	0
1	1	1	3
1	1	1	3
1	1	1	3
0	0	0	0
1	1	1	3
1	1	1	3
1	1	1	3
0	0	0	0
0	0	0	0
1	1	1	3
0	0	0	0
1	1	1	3
0	0	0	0
0	0	0	0
0	0	0	0
1	1	1	3
1	1	1	3
1	1	1	3
1	1	1	3
1	1	1	3
1	1	1	3
0	0	0	0
0	0	0	0
1	1	1	3
1	1	1	3
1	1	1	3
1	1	1	3
1	1	1	3
0	0	0	0
0	0	0	0
0	0	0	0
0	0	0	0
0	0	0	0
0	0	0	0
0	0	0	0
0	0	0	0

0	0	0	0
0	0	0	0
0	0	0	0
0	0	0	0
0	0	0	0
0	0	0	0
0	0	0	0
0	0	0	0
1	1	1	3
1	1	0	2
1	1	1	3
1	1	1	3
1	1	1	3
1	1	1	3
0	0	0	0
1	1	0	2
1	1	0	2
1	1	1	3
1	0	1	2
0	0	1	1
1	1	1	3
0	0	0	0
1	1	1	3
1	1	1	3
0	0	0	0
0	0	0	0
0	0	1	1
1	1	1	3
0	0	0	0
0	0	0	0
1	1	0	2
1	1	1	3
0	0	1	1
0	0	1	1
0	0	1	1
1	0	1	2
0	0	0	0
1	0	1	2
0	0	1	1
0	0	0	0
0	0	0	0
0	0	0	0
0	0	1	1
0	0	0	0
0	0	1	1
0	0	0	0
0	0	0	0
0	0	0	0
0	0	0	0
0	0	0	0
1	1	1	3
0	0	0	0
1	1	1	3
0	0	0	0
0	0	0	0
1	1	1	3
1	1	1	3
1	1	1	3
1	1	1	3

0	0	0	0
1	1	1	3
1	1	1	3
0	0	1	1
0	0	0	0
0	0	0	0
0	0	0	0
0	0	0	0
0	0	0	0
1	1	1	3
1	1	1	3
0	0	0	0
0	0	0	0
1	1	1	3
0	0	0	0
0	1	1	2
0	1	1	2
0	0	0	0
1	1	1	3
0	0	0	0
0	0	0	0
1	1	1	3
0	0	0	0
1	1	1	3
0	0	1	1
1	1	1	3
1	1	1	3
0	0	0	0
0	0	1	1
1	1	1	3
1	0	0	1
1	1	1	3
1	1	1	3
0	0	1	1
0	1	1	2
1	1	1	3
0	0	0	0
1	0	1	2
0	0	0	0
1	1	1	3
1	1	1	3
0	0	1	1
0	0	0	0
0	0	1	1
0	0	0	0
1	1	0	2
1	0	1	2
1	1	1	3
0	0	1	1
0	0	0	0
0	0	1	1
0	0	0	0
0	0	1	1
0	0	1	1
1	1	1	3
0	0	0	0
0	0	0	0
0	0	0	0
1	0	0	1

1	1	1	3
0	0	0	0
0	0	0	0
1	1	1	3
0	0	0	0
1	1	1	3
1	1	1	3
1	1	1	3
1	1	1	3
0	1	1	2
0	0	0	0
0	0	0	0
0	0	0	0
0	0	1	1
0	0	0	0
0	1	1	2
0	0	0	0
1	1	1	3
0	0	0	0
1	1	1	3
0	0	0	0
0	0	0	0
1	1	1	3
1	1	1	3
1	1	1	3
0	0	0	0
1	1	1	3
1	1	1	3
0	0	0	0
1	1	1	3
1	1	1	3
0	0	0	0
1	1	1	3
1	1	1	3
1	1	1	3
1	1	1	3
1	1	1	3
1	1	1	3
1	1	1	3
0	0	0	0
1	1	1	3
0	0	0	0
0	0	0	0
0	0	1	1
1	1	1	3
1	1	1	3
1	1	1	3
0	0	0	0
0	0	0	0
0	1	1	2
1	0	0	1
1	1	1	3
0	1	1	2
1	0	0	1
1	1	1	3
1	1	1	3
1	1	0	2
1	1	1	3
0	0	1	1

0	0	0	0
0	0	0	0
0	0	0	0
0	0	0	0
0	0	0	0
0	0	1	1
1	1	1	3
1	1	1	3
0	1	1	2
0	0	0	0
0	0	0	0
0	0	0	0
0	0	0	0
0	0	0	0
1	1	1	3
0	0	0	0
1	1	1	3
1	1	1	3
0	0	0	0
1	1	1	3
0	0	0	0
0	1	1	2
0	1	1	2
0	0	0	0
0	0	0	0
0	1	1	2
0	0	0	0
0	0	0	0
0	0	0	0
0	0	0	0
1	1	1	3
1	1	1	3
1	1	1	3
0	0	0	0
1	1	1	3
1	1	1	3
1	1	1	3
0	0	0	0
0	0	0	0
0	0	0	0
1	1	1	3
0	1	1	2
1	0	0	1
0	1	1	2
1	0	0	1
0	1	1	2
1	0	0	1
0	1	1	2
1	0	0	1
0	1	1	2
1	0	0	1
0	0	0	0
0	0	0	0
0	0	0	0
0	1	1	2
1	1	1	3
1	1	1	3
1	1	1	3
1	1	1	3

0	0	0	0
1	1	1	3
1	1	1	3
1	1	1	3
0	0	0	0
1	0	0	1
0	0	0	0
1	1	1	3
1	1	1	3
0	0	0	0
0	0	0	0
1	1	1	3
1	1	1	3
0	0	0	0
1	1	1	3
1	1	1	3
1	1	1	3
0	0	1	1
0	0	0	0

[redacted]

(R = 20°C)

R1	R2	R3	sum
1	1	1	3
0	0	0	0
1	1	1	3
1	1	1	3
1	1	0	2
1	1	1	3
1	1	1	3
0	0	0	0
1	1	1	3
0	0	0	0
1	1	1	3
1	1	0	2
1	1	1	3
1	1	0	2
1	1	1	3
1	1	1	3
0	0	0	0
1	1	1	3
1	1	1	3
1	1	1	3
0	0	0	0
0	0	0	0
1	1	1	3
1	1	1	3
0	0	0	0
1	1	1	3
0	0	0	0
1	1	1	3
0	0	0	0
0	0	0	0
0	0	0	0
1	1	1	3
0	0	0	0

0	0	0	0
0	0	0	0
0	0	0	0
1	0	1	2
0	0	0	0
0	0	0	0
0	0	0	0
1	1	1	3
0	0	0	0
1	1	1	3
1	1	1	3
0	0	1	1
0	0	0	0
1	1	1	3
1	1	1	3
0	0	0	0
0	0	0	0
0	0	0	0
0	0	1	1
1	1	1	3
0	0	0	0
0	0	0	0
0	0	0	0
1	1	1	3
0	0	1	1
1	1	1	3
0	0	0	0
0	0	0	0
1	1	1	3
1	0	0	1
1	1	1	3
0	0	0	0
0	0	0	0
0	0	0	0
1	1	1	3
1	1	1	3
1	1	1	3
1	0	1	2
1	1	1	3
0	1	1	2
0	0	0	0
1	1	1	3
1	1	1	3
0	0	0	0
1	1	1	3
0	0	0	0
0	0	0	0
0	0	0	0
0	0	0	0
1	1	1	3
0	0	0	0
0	0	0	0
0	0	0	0
0	0	0	0
0	0	0	0
1	1	1	3
0	0	0	0
0	0	0	0
0	0	0	0

0	0	0	0
0	0	0	0
0	0	0	0
1	1	1	3
1	1	1	3
1	1	1	3
0	0	0	0
0	0	1	1
1	1	1	3
1	1	1	3
1	1	1	3
1	1	1	3
0	0	0	0
1	1	1	3
1	1	1	3
0	0	0	0
0	0	0	0
0	0	0	0
1	1	1	3
1	1	0	2
0	0	0	0
1	1	1	3
0	0	0	0
0	0	0	0
1	1	0	2
0	0	0	0
0	0	0	0
1	0	0	1
1	1	0	2
1	1	1	3
0	0	0	0
0	0	0	0
0	0	0	0
0	0	0	0
0	0	0	0
0	0	0	0
1	1	0	2
0	0	0	0
0	0	0	0
0	0	1	1
1	1	1	3
1	0	1	2
0	0	0	0
0	0	0	0
1	0	0	1
0	0	0	0
0	0	0	0
1	0	0	1
1	0	0	1
0	0	0	0
1	1	0	2
1	0	0	1
0	0	0	0
0	0	0	0
0	0	0	0
0	0	0	0
0	0	0	0
0	0	0	0
0	0	0	0

1	1	0	2
1	1	1	3
0	0	0	0
1	1	1	3
0	0	0	0
1	0	0	1
0	0	0	0
0	0	0	0
0	0	0	0
0	0	0	0
0	0	0	0
0	0	0	0
0	0	0	0
0	0	0	0
0	0	0	0
0	0	0	0
0	0	0	0
0	0	0	0
1	1	0	2
0	0	0	0
0	0	0	0
1	0	0	1
1	1	1	3
1	1	1	3
1	1	1	3
1	1	1	3
0	0	0	0
1	1	0	2
0	0	0	0
1	1	0	2
0	0	0	0
0	0	0	0
1	1	1	3
0	0	1	1
0	0	0	0
0	0	0	0
1	1	1	3
1	1	1	3
1	1	1	3
0	0	0	0
0	0	0	0
1	1	1	3
0	0	0	0
1	1	0	2
1	1	1	3
0	0	0	0
0	0	0	0
1	1	1	3
0	0	0	0
1	1	1	3
0	0	0	0
0	0	0	0
0	0	0	0
0	0	0	0
0	0	0	0
0	0	0	0
0	0	0	0
0	0	0	0
1	1	1	3

0	0	0	0
0	0	0	0
1	1	1	3
0	0	0	0
0	0	0	0
0	0	1	1
0	0	0	0
0	0	0	0
0	0	0	0
0	0	0	0
0	0	0	0
1	1	1	3
1	1	1	3
0	1	1	2
1	1	1	3
0	0	0	0
1	1	0	2
1	1	1	3
0	0	0	0
1	1	1	3
0	0	0	0
1	1	1	3
1	1	1	3
1	1	0	2
1	0	1	2
0	0	1	1
0	0	0	0
0	0	1	1
1	1	0	2
0	0	1	1
0	0	0	0
0	0	0	0
0	0	0	0
0	0	1	1
0	0	1	1
0	0	0	0
0	0	0	0
0	0	0	0
0	0	1	1
1	1	1	3
0	0	1	1
1	0	0	1
0	0	0	0
0	0	0	0
0	0	0	0
0	0	0	0
0	0	0	0
0	0	0	0
0	0	0	0
0	0	0	0
0	0	0	0
1	1	1	3
1	1	1	3
1	1	1	3
0	0	0	0
0	0	0	0
0	0	0	0
0	0	0	0
1	1	1	3

0	0	1	1
1	1	1	3
1	1	1	3
0	0	0	0
1	1	1	3
1	0	0	1
0	0	0	0
1	1	1	3
0	0	0	0
0	0	1	1
1	1	1	3
0	0	0	0
1	1	1	3
1	0	0	1
1	1	1	3
0	0	0	0
1	1	1	3
0	0	0	0
1	1	1	3
0	0	0	0
0	0	0	0
1	1	1	3
1	1	1	3
1	1	1	3
0	0	0	0
0	0	0	0
0	0	0	0
1	1	1	3
0	0	0	0
1	1	1	3
0	0	0	0
0	0	0	0
0	0	0	0
0	0	0	0
1	1	1	3
1	1	1	3
0	0	0	0
1	1	1	3
0	0	0	0
0	0	0	0
0	0	0	0
1	1	1	3
0	0	0	0
0	0	0	0
0	1	0	1
0	0	0	0
1	1	1	3
0	0	0	0
0	0	0	0
0	0	0	0
1	1	0	2
0	0	1	1
1	1	1	3
0	0	0	0
0	0	0	0
0	0	0	0
0	0	0	0
0	0	0	0
1	1	0	2

0	0	0	0
0	0	0	0
0	0	0	0
0	0	0	0
0	0	0	0
0	0	0	0
0	0	0	0
0	0	0	0
1	1	1	3
0	0	0	0
0	0	0	0
0	0	0	0
1	1	1	3
0	0	0	0
0	0	0	0
0	0	0	0
1	1	1	3
0	0	0	0
1	1	1	3
0	0	0	0
0	0	0	0
1	1	1	3
0	0	0	0
1	1	0	2
0	0	0	0
1	1	0	2
1	1	0	2
0	0	0	0
1	1	1	3
0	0	0	0
1	1	1	3
1	0	0	1
0	0	0	0
0	0	1	1
1	1	1	3
1	1	1	3
1	1	1	3
1	1	1	3
0	0	0	0
1	1	1	3
0	0	0	0
0	0	0	0
1	1	1	3
0	0	0	0
0	0	0	0
0	0	0	0
0	0	0	0
0	0	0	0
0	0	0	0
0	0	0	0
0	0	0	0
0	0	0	0
0	0	0	0
0	0	0	0
1	1	1	3
0	0	0	0
1	1	1	3
1	1	1	3
1	1	1	3

0	0	0	0
1	1	1	3
1	1	1	3
1	1	1	3
0	0	0	0
1	1	1	3
1	1	1	3
1	1	1	3
0	0	1	1
0	0	0	0
1	1	1	3
0	0	0	0
0	0	0	0
0	0	0	0
0	0	0	0
1	1	0	2
0	0	0	0
0	0	0	0
1	1	1	3
0	0	0	0
0	0	0	0
1	1	1	3
0	0	0	0
1	1	1	3
0	0	0	0
0	0	0	0
1	1	0	2
0	0	0	0
0	1	0	1
1	1	0	2
1	1	0	2
1	1	0	2
1	1	0	2
1	1	0	2
0	0	0	0
1	1	0	2
0	0	0	0
1	1	0	2
0	0	0	0
0	0	0	0
0	0	0	0
1	1	0	2
1	1	0	2
1	1	0	2
0	0	0	0
1	1	0	2
1	1	0	2
1	1	0	2
1	0	0	1
0	0	0	0
0	0	0	0
1	0	0	1
0	0	0	0
0	0	0	0
0	0	0	0
0	0	0	0
0	0	0	0
0	0	0	0
0	1	0	1

0	0	0	0
1	1	0	2
1	1	0	2
0	0	0	0
0	0	0	0
0	0	0	0
0	0	0	0
1	1	0	2
1	1	0	2
0	0	0	0
1	1	0	2
0	1	0	1
0	0	0	0
0	1	0	1
0	0	0	0
0	0	0	0
1	1	0	2
1	1	0	2
1	1	0	2
0	0	0	0
1	1	0	2
1	1	0	2
0	0	0	0
0	0	0	0
0	0	0	0
0	0	0	0
1	1	0	2
1	1	0	2
0	0	0	0
0	0	0	0
0	0	0	0
0	0	0	0
0	0	0	0
0	0	0	0
0	0	0	0
1	1	0	2
0	0	0	0
1	1	0	2
0	1	1	2
0	0	0	0
0	1	1	2
1	1	1	3
1	1	1	3
1	1	1	3
1	1	1	3
1	1	1	3
0	0	0	0
0	0	0	0
1	1	1	3
0	0	0	0
0	0	0	0
0	0	0	0
0	0	0	0
0	0	1	1
0	0	0	0
0	0	0	0
0	0	0	0
1	1	1	3
0	0	0	0

0	0	0	0
0	0	0	0
0	0	0	0
0	0	0	0
0	0	0	0
0	0	0	0
0	1	0	1
1	1	1	3
1	1	1	3
1	1	1	3
1	1	1	3
0	0	0	0
1	1	1	3
1	1	1	3
0	0	0	0
1	1	1	3
0	0	0	0
1	1	1	3
0	0	0	0
0	0	0	0
0	0	0	0
0	0	0	0
1	1	1	3
0	0	0	0
0	0	0	0
1	1	1	3
1	1	1	3
1	1	1	3
0	0	0	0
0	0	0	0
1	1	1	3
0	0	0	0
0	0	0	0
1	1	1	3
0	0	0	0
0	0	0	0
0	0	0	0
0	0	0	0
1	1	1	3
1	1	1	3
0	0	0	0
1	1	1	3
1	1	1	3
0	0	0	0
0	0	0	0

5271
#RIF!
